# Skeletal microstructures of cheilostome bryozoans (phylum Bryozoa, class Gymnolaemata): crystallography and secretion patterns

**DOI:** 10.1007/s42995-024-00233-1

**Published:** 2024-06-07

**Authors:** Christian Grenier, Erika Griesshaber, Wolfgang Schmahl, Björn Berning, Antonio G. Checa

**Affiliations:** 1 Departamento de Estratigrafía y Paleontología, Universidad de Granada, 18071 Granada, Spain; 2 Instituto Andaluz de Ciencias de La Tierra, CSIC-Universidad de Granada, 18100 Armilla, Spain; 3 Department of Earth and Environmental Sciences, Ludwig-Maximilians Universität, 80333 Munich, Germany; 4 Institute for Geology, University of Hamburg, 20146 Hamburg, Germany

**Keywords:** Biomineralization, Bryozoan, Skeleton, Calcite, Aragonite, Electron backscatter diffraction

## Abstract

Gymnolaemata bryozoans produce CaCO_3_ skeletons of either calcite, aragonite, or both. Despite extensive research, their crystallography and biomineralization patterns remain unclear. We present a detailed study of the microstructures, mineralogy, and crystallography of eight extant cheilostome species using scanning electron microscopy, electron backscatter diffraction, atomic force microscopy, and micro-computed tomography. We distinguished five basic microstructures, three calcitic (tabular, irregularly platy, and granular), and two aragonitic (granular-platy and fibrous). The calcitic microstructures consist of crystal aggregates that transition from tabular or irregularly platy to granular assemblies. Fibrous aragonite consists of fibers arranged into spherulites. In all cases, the crystallographic textures are axial, and stronger in aragonite than in calcite, with the c-axis as the fiber axis. We reconstruct the biomineralization sequence in the different species by considering the distribution and morphology of the growth fronts of crystals and the location of the secretory epithelium. In bimineralic species, calcite formation always predates aragonite formation. In interior compound walls, growth proceeds from the cuticle toward the zooecium interior. We conclude that, with the exception of tabular calcite, biomineralization is remote and occurs within a relatively wide extrapallial space, which is consistent with the inorganic-like appearance of the microstructures. This biomineralization mode is rare among invertebrates.

**Supplementary Information:**

The online version contains supplementary material available at 10.1007/s42995-024-00233-1.

## Introduction

Bryozoans comprise an entire phylum of aquatic, colonial animals that occur worldwide in fresh and marine waters, from shallow to deep waters (Ryland 1977; Taylor 2020). The colonies are made of clonal units, called zooids, which differentiate during their development (ontogeny) into various types (polymorphs) according to their function in the colony: feeding autozooids, structural kenozooids, defensive avicularia, and brooding chambers called gonozooids (see Ryland 1977; Taylor 2020) for a biological introduction to the phylum). The total number of living bryozoan species is estimated at ~ 6500, distributed among the classes Gymnolaemata (> 85% of the extant bryozoan species), Phylactolaemata (< 100 species), and Stenolaemata (654 species) (WoRMS Editorial Board 2024).

More than 98% of bryozoans live in seawater and secrete calcium carbonate skeletons, which makes them an important fossil group (Hageman et al. 2003; O’Dea et al. 2011; Taylor and Allison 1998). The non-marine bryozoans (Phylactolaemata) are unmineralized. The order Cheilostomatida, which appeared in the Late Jurassic (Taylor 1994) and diversified from the mid-Cretaceous on (Jablonski et al. 1997), became the current, dominant group of bryozoans (Taylor 2020).

Cheilostomatida is the major order of gymnolaemates, comprising more than 94% of the species. They all live in marine environments (except a few species that occur in brackish waters) and produce mineralized skeletons that are covered by an organic layer, the cuticle. The cuticle forms at the growing edge of the colony where newly budded zooids originate. It is secreted by an epithelium consisting of palisade, fusiform, and vesicular cells, and serves as a template for the nucleation of calcium carbonate crystals during the early stages of skeleton formation (see fig. 1 in Tavener-Smith and Williams 1972).

**Fig. 1:**
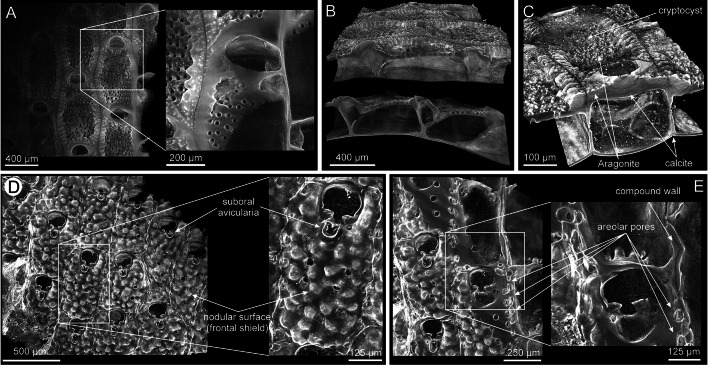
Micro-CT images of the encrusting colonies of *Calpensia nobilis* (**A**–**C**) and *Schizomavella cornuta* (**D**–**E**). **A** Frontal view of the colony fragment. The zooids are arranged in a single layer; they are aligned in rows and offset with respect to the zooids of the neighboring rows. The orifices display a semicircular shape and the cryptocyst, which lies below the frontal membrane in the living organism, is perforated by pores. The interior of the chambers of the zooid is progressively exposed toward the bottom (zoomed view of the framed area), revealing the thin interior walls. **B** Two lateral views of the same fragment sectioned at different depths. The unilaminar encrusting colony structure can be seen. **C** Oblique view of an isolated zooid. There is a slight contrast between the calcitic interior walls (darker) and the aragonitic cryptocyst (lighter) (indicated). **D** Frontal view of a colony fragment. The orifices display a semicircular shape and all zooids have small sub-oral avicularia. The frontal shield exhibits a nodular surface (close-up view to the right). The zooids are slightly offset to each other. **E** Fragment sectioned slightly oblique to the frontal surface; the areolar pores can be seen along the interior compound walls

All cheilostomes have a characteristic box-shaped zooidal chamber. They have three types of zooidal walls: basal, vertical (parallel or transverse to the elongation axis), and frontal. Basal and vertical walls are usually calcified, except for communication windows (porous septulae) or special modes of attachment to the substrate. The greatest differences between species are found in the structure of the frontal wall, which, in the informal group Anasca, may be either wholly membranous (or underlain by a calcified cryptocyst in some taxa) or calcified to form a frontal shield (in the informal group Ascophora) (Dick et al. 2009). A second diagnostic feature to consider is whether the walls are interior walls (internal partitions where secretory epithelia are found on both surfaces) or exterior walls (secretory epithelium on one side and the cuticle on the opposite side). Additionally, two external walls separating adjacent zooids, with their cuticles placed side by side in the center, are defined as compound walls (Taylor et al. 2015).

Skeletons are made of two calcium carbonate polymorphs: calcite and aragonite. Some species are either entirely calcitic or, much rarely, entirely aragonitic, while others secrete both polymorphs (bimineralic) (e.g., Lowenstam 1954; Sandberg 1977; Smith et al. 2006; Taylor et al. 2009). Numerous studies performed on many species (summarized in Taylor et al. 2009) have demonstrated a strong latitudinal pattern: calcitic bryozoans are dominant at high latitudes (around the polar circles) while moving toward the equator the number of aragonitic and bimineralic species increases until reaching a proportion similar to the calcitic ones.

Other studies based on scanning electron microscopy (SEM), electron microprobe (EMP), and Raman spectroscopy (Benedix et al. 2014; Taylor et al. 2008) have determined the location of both calcium carbonate polymorphs within the skeleton of some bimineralic bryozoans. Aragonite is restricted to the outer layer of the frontal shield (in ascophorans) or the cryptocyst (in anascans), while the rest of the skeletal walls are usually made of calcite. Occasionally, aragonite is located in adventitious avicularia in *Odontionella cyclops* and *Stylopoma inchoans*, or in the outermost part of the basal walls in *Pentapora foliacea* (Taylor et al. 2008).

Regarding the microstructures of cheilostomes, the majority of studies have focused on bimineralic species (Taylor et al. 2015). The first studies based on SEM images and X-ray diffraction were performed on the genus *Metrarabdotos* (Boardman and Cheetham 1969; Cheetham et al. 1969). According to their results, a primary calcitic “lamellar layer” constituted the basal and the interior vertical walls as well as the inner layer of the frontal shields, while a secondary aragonitic “fibrous or wavy-lamellar layer” formed the outermost layer of the frontal shields. Later, in an in-depth study with fossil and recent specimens, Sandberg (1971) recognized the same aragonitic microstructure, which he named “transverse fibrous”; Sandberg described six different calcitic microstructures, grouped into “parallel layers” (lamellar, massive internal, fibrous, and crystal stacks) and “transverse layers” (columnar or “cell-mosaic” and transverse fan). More recently, Weedon and Taylor (2000) recognized seven fabric suites in sixteen extant anascan (with primitive organization) cheilostome species. All these microstructures can be grouped into both “platy or lamellar-like microstructures”, where the plates are arranged into irregular lamellae and “perpendicular fibrous aragonitic microstructure”, with fibers arranged into spherulites in the frontal shield (Benedix et al. 2014; Sandberg 1977; Smith and Girvan 2010).

In entirely calcitic cheilostomes, the same platy layer was found by Bader and Schäfer (2004), but, additionally, they described a secondary inner layer defined as “densely packed crystallites” (i.e., granular layer). There have been fewer studies done exclusively on aragonitic bryozoans. This is most likely due to their lower abundance (notable examples are the free-living genera *Cupuladria* and the hermit crab-symbiont *Hippoporidra*; see Taylor et al. 2015). However, their microstructures are similar to those found in bimineralic bryozoans: elongated thin fibers arranged into spherulites.

Despite the relevance of cheilostome bryozoans as calcifiers, only one study has dealt with the crystallography of the microstructures. Examining the bimineralic *Anoteropora latirostris*, Jacob et al. (2019) determined by SEM-EBSD that calcite grain aggregates showed a stronger texture (“5–7,4 multiples of uniform distribution”) than those of aragonite (“2,4 multiples of uniform distribution”). Both calcite and aragonite aggregates displayed a significant intragrain misorientation (identified by Jacob et al. (2019), as “mesocrystals”) and ~ 45% of aragonite grains were twinned on the {110} plane. However, regarding cheilostome bryozoans, there is a lack of in-depth studies that elucidate the repertoire and crystallography of microstructures, in a way similar to those conducted on other calcifying groups, such as molluscs, brachiopods, corals, and foraminifera (Checa et al. 2021; Coronado et al. 2019; Crippa et al. 2020; Simonet Roda et al. 2022; Yin et al. 2021).

Here, we analyze the mineralogy, crystallography, and distribution of the calcitic and aragonitic microstructures present in eight cheilostome bryozoan species by SEM, energy-dispersive spectroscopy (EDS), and electron backscattered diffraction (EBSD). Additional data were obtained with atomic force microscopy (AFM) and micro-computed tomography (Micro-CT). Taking into account the growth directions of crystal aggregates and the morphology and distribution of growth lines, we unravel the sequence of biomineralization in the different species.

## Materials and methods

### Material

Eight species of cheilostomate bryozoans were analyzed: the anascan *Calpensia nobilis* (Esper, 1796), fam. Microporidae (though the recent molecular studies of Orr et al. 2022, and Grant et al. 2023 suggest that *Calpensia* is closely related to Thalamoporellidae and Steginoporellidae) and the ascophorans *Schizobrachiella sanguinea* (Norman, 1868), fam. Schizoporellidae, *Rhynchozoon neapolitanum* (Gautier, 1962), fam. Phidoloporidae, *Schizoretepora serratimargo* (Hincks, 1886), fam. Phidoloporidae, *Pentapora fascialis* (Pallas, 1766), fam. Bitectiporidae, *Adeonella pallasii* (Heller, 1867), fam. Adeonidae, *Schizomavella cornuta* (Heller, 1867), fam. Bitectiporidae, and *Smittina cervicornis* (Pallas, 1766), fam. Smittinidae. All these species are presently valid according to WoRMS Editorial Board (2024). Samples were collected at depths between 3 and 20 m in the Adriatic Sea (Korčula and Lombarda Island, Croatia) in 2008 and stored in 97% ethanol. Provenance and collection data are summarized in Supplementary Fig. S1.

### Micro-computed tomography (micro-CT)

Fragments of colonies of four species (*Calpensia nobilis, Schizoretepora serratimargo*, *Schizomavella cornuta*, and *Smittina cervicornis*) were selected for micro-CT analyses. The scans were performed in an X-ray microtomograph Xradia 510 VERSA Zeiss (CIC, UGR). The fragments were fixed to the tip of a needle holder with glue and calibrated with the following parameters: 80 kV acceleration voltage, 7W power, 75 μA beam current, and 4 × magnification objective. The binning used was “bin 1” for the CDD camera detector, with a voxel size of 0.9951 µm. The total number of projections was 3201. The distance from the source to the sample was 25 mm, and the distance from the detector to the sample was 60 mm. The exposure time (per projection) and the source filter were adjusted for each sample: 14 s and LE2 filter for *C. nobilis*, 20 s and LE2 filter for *S. serratimargo*, 24 s and LE3 filter for *S. cornuta*, and 22 s and LE4 filter for *S. cervicornis*. For center shift and beam hardening effect corrections, images were processed using Reconstructor Scout and Scan™ (Zeiss, Oberkochen, Germany). We used 3D image analysis Dragonfly Pro™ (Object Research System, slice registration method SSD) for advanced post-processing.

### Scanning electron microscopy (SEM)

Fragments of each species were cleaned by immersion in commercial bleach (~ 5% active chlorine) for 2 h in a stirring set. Then, the bleach solution was removed by several sonicated washes in ultrapure water, for 2–3 min each. Once oven-dried at 40 °C for 24 h, some fragments were selected for observation, whereas others were embedded in epoxy resin (EpoFix, Struers). After 48 h of hardening, each sample section was exposed by successive grinding steps with 360, 600, 1200, and 3000 (ANSI/CAMI US grit numbers) electroplated diamond discs. Subsequently, the surfaces were polished with high-density wool felt pad discs (adding first 1 µm, followed by 0.25 µm polycrystalline diamond suspension, Struers), until reaching a mirror surface. A Hi-Tech Diamond polishing machine (All-U-Need model) was used both for grinding and polishing. Finally, an etching and decalcifying solution (2.5% glutaraldehyde, 0.25 mmol/L HEPES buffer, and 0.05 mmol/L EDTA) was applied directly to the exposed surfaces for 1 min in a stirring module. SEM observations were performed after carbon coating (Emitech K975X carbon evaporator) using secondary electron (SE) and back scatter electron (BSE) detectors in a field emission SEM FEI QemScan 650 F, a Helios Nanolab 650, and a Carl Zeiss SMT AURIGA Crossbeam Station. All the equipment was housed in the Centro de Instrumentación Científica (CIC, University of Granada, UGR) and in the Servicios Centrales de Apoyo a la Investigación (SCAI, University of Málaga, UMA).

### Electron backscattered diffraction (EBSD)

EBSD maps (61) were performed on the eight species. The same samples prepared for SEM observation were finished with a manual etch-polishing step, applying colloidal alumina for 3 min. The samples were coated with 4–6 nm of carbon (Leica EM ACE200). Measurements were taken on a Hitachi SU5000 field emission SEM, equipped with an Oxford Instruments NordlysNano II EBSD detector. The SEM was operated at 20 kV, and Kikuchi patterns were indexed with the CHANNEL 5 HKL software. EBSD measurements were performed in step increments between 200 and 500 nm.

The EBSD diffraction measurements are presented as band contrast measurement images and as Inverse Pole Figure (IPF) maps. White or dark regions in the band contrast maps correspond to higher or weaker signal strength, respectively, of the EBSD Kikuchi diffraction patterns. The IPF maps are color-coded crystal orientation images, where similar colors indicate similar orientations. The term “texture” relates to the orientation of the crystallographic axes within a material. The texture is represented by pole figures (PFs) whose spatial orientation matches that of the corresponding EBSD map. The PFs are presented either as individual data points or, in the contoured version, as the densities of poles. In the latter case, we use the lowest possible degree for half-width (5°) and cluster size (3°). The half-width controls the extent of the spread of the poles over the surface of the project sphere. A cluster comprises data with the same orientation. We also provide the multiple of uniform distribution (MUD) values. A high MUD value indicates high crystal co-orientation, while low MUD values point to random orientation or low crystal co-orientation. MUD > 700 indicates a single crystal nature.

## Atomic force microscope (AFM)

Colony fragments of *Rhynchozoon neapolitanum*, *Pentapora fascialis*, and *Smittina cervicornis* were cleaned by immersion in commercial bleach (∼5% active Cl, 8 h). The bleach solution was then removed by 2 to 3 washes in MilliQ water by sonication, for 2–3 min each. Once oven-dried at 40 °C for 24 h, samples were reduced to small fragments, oriented, and placed on flat metal discs for AFM observation, so that the external surfaces and walls sections were exposed. Additionally, the samples of these species prepared for SEM (embedded in resin and polished as explained above) were analyzed. An AFM Park Systems NX20 equipped with a cantilever MikroMasch ACTA (*K* = 40 N/m, *F* = 320 kHz) (CIC, UGR) was used in Tapping and PinPoint modes to record height, amplitude, and phase signals. Images were obtained with Smart Scan v12 and processed using XEI software (4.3.0. Build2, Park Systems).

## Results

### Colony structure and zooid arrangement.

**Fig. 2:**
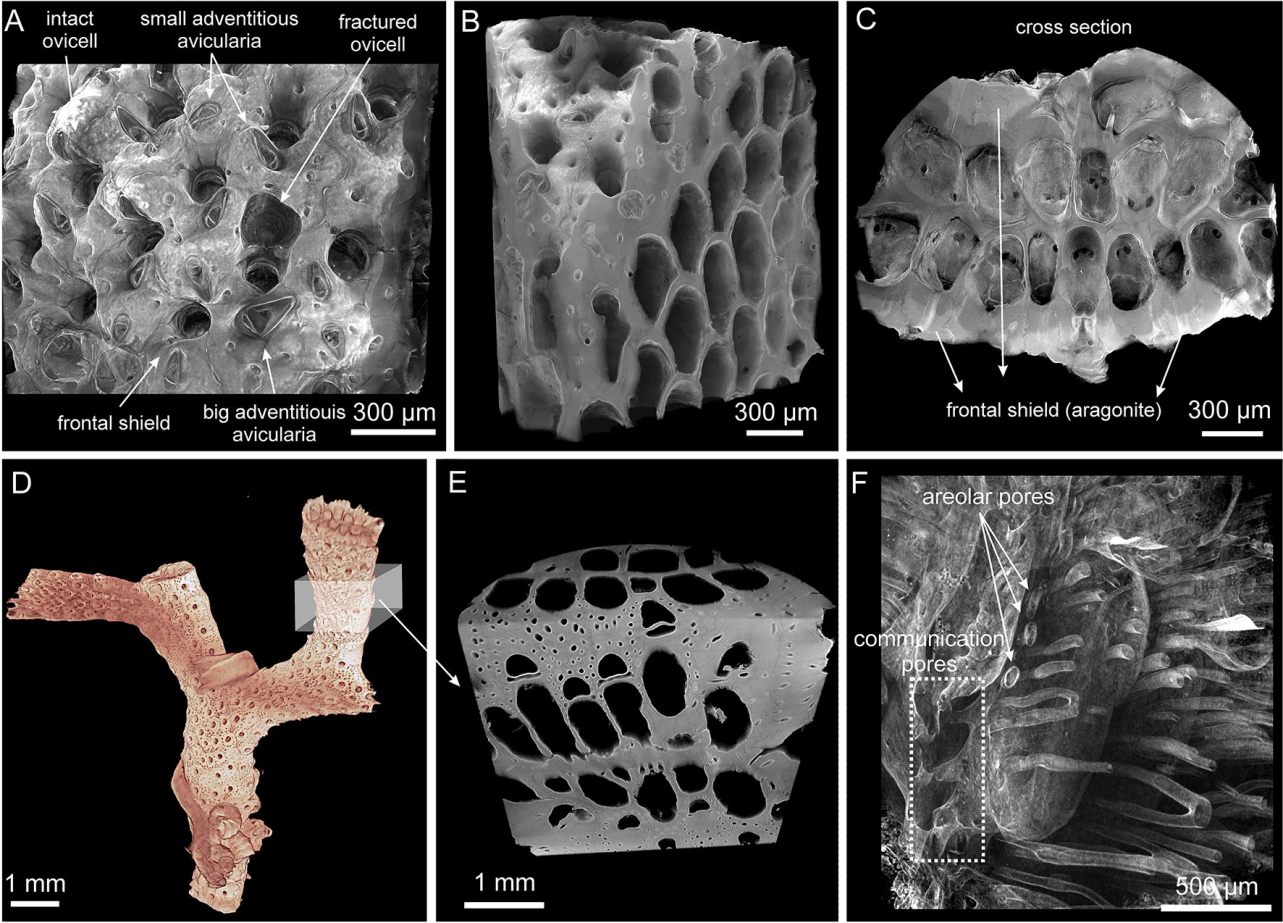
Micro-CT images of the erect ramose colonies of *Schizoretepora serratimargo* (**A**–**C**) and *Smittina cervicornis* (**D**–**F**)*.* **A** Frontal view of the colony fragment. Different polymorphic zooids can be observed (autozooids and avicularia). **B** Same fragment as in A sectioned longitudinally, exposing the interior of the zooids and the thick interior vertical walls (compound walls). The zooids are arranged in a zigzag pattern. **C** Cross-sectional view, showing the double-layer arrangement of the zooids. **D** Erect-branched colony. **E** Close-up of a branch sectioned along two perpendicular planes, demonstrating the zooid disposition and the thick external frontal shields. **F** Image of the inner volume occupied by the zooidal polypides, revealing the areolar channels oriented toward the frontal shield

The bryozoans were analyzed from either encrusting unilaminar (e.g., *Calpensia nobilis* and *Schizomavella cornuta*; Fig. 1A–E) or erect ramose bilaminar colonies (e.g., *Schizoretepora serratimargo* and *Smittina cervicornis*; Fig. 2A–F). In the encrusting colonies, the basal wall attaches directly to the substratum (Fig. 1A, B) while in the erect colonies, the zooids are disposed in a bilaminar layer, with back-to-back basal walls (Fig. 2C, E).

Some of the species studied exhibit a well-developed polymorphism. For instance, avicularia can be distinguished in both *S. cornuta* and *S. serratimargo*. *S. cornuta* has only small sub-oral avicularia (Fig. 1D) and *S. serratimargo* has numerous adventitious avicularia, some of which are larger than others (Fig. 2A). Intact and fractured ovicells (calcified brooding capsules for the embryos) can be found distally from the orifice in some autozooids (rounded and darker depressions in Fig. 2A).

An example of the inner volume occupied by the zooids and the funicular system is shown for *S. cervicornis* (Fig. 2F). The areolar pores connect the inner zooidal coelom with the hypostegal coelom (between the frontal shield and the frontal cuticle). Additionally, the communication pores that cross the compound lateral walls of two neighboring zooids are highlighted in the framed area of Fig. 2F.

### Mineralogy

The distribution of calcium carbonate polymorphs (calcite and aragonite) was determined by EBSD phase maps and Mg/Sr EDX maps (Supplementary Figs. S2-S4). We grouped the eight cheilostome species into: (1) bimineralic (*Schizobrachiella sanguinea*, *Calpensia nobilis*, *Schizoretepora serratimargo*, *Pentapora fascialis*, and *Adeonella pallasii,* Supplementary Fig. S2), (2) calcitic (*Smittina cervicornis, *Supplementary Fig. S3), and (3) predominantly aragonitic (*Rhynchozoon neapolitanum* and *Schizomavella cornuta,* Supplementary Fig. S4). In bimineralic bryozoans, calcite constitutes the main structures of the zooecium: interior vertical and compound walls and the first basal layers of the frontal shield (or cryptocyst in *C. nobilis*), while aragonite is only present in the outermost layer of the frontal shield (or cryptocyst in *C. nobilis*). We identified five main microstructures: three calcitic (tabular calcite, irregularly platy calcite, and granular calcite) and two aragonitic (fibrous aragonite and granular-platy aragonite).

### Microstructures

#### Bimineralic bryozoans

**Calcite microstructures**

**Fig. 3:**
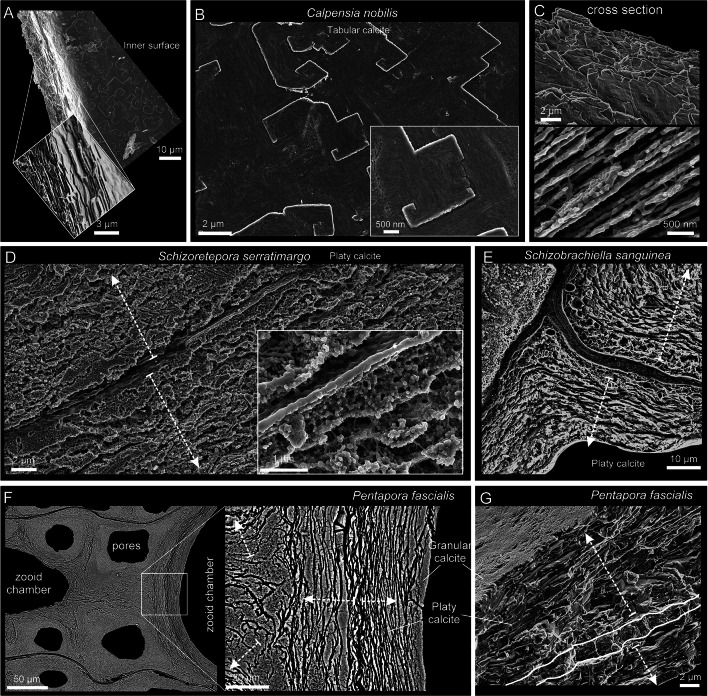
SEM images of the tabular (**A**–**C**) and platy (**D**–**G**) calcites. **A**–**C** *Calpensia nobilis*. **A** Fragment of interior vertical and basal walls, composed entirely of tabular calcite (inset) in an early stage of development. **B** Detailed view of an unaltered inner surface. The spiral-shaped polygonal tablets cover the entire surface. The surface nano-roughness and the growth lines are visible (inset). **C** Cross-section of a wall showing the stacking of tablets, which produces a lamellar arrangement. The organic threads between the crystals are visible in the bottom image.** D** Polished and slightly decalcified compound wall of *Schizoretepora serratimargo* made of irregularly platy calcite. The crystals grow from the cuticle inwards in both directions (arrows). The inset shows the organic threads that surround the crystals. **E** Polished-etched section through compound walls of *Schizobrachiella sanguinea*, showing the irregular and sinuous outlines of the laminae of the irregularly platy calcite. The lamellar crystals are arranged parallel to the inner surface, adapting tightly to the curvature at the corners. Dashed arrows indicate the growth directions of the layers. **F**, **G** *Pentapora fascialis*. **F** Polished section along two zooid chambers. The zoomed area shows the irregularity of the platy calcite. The dashed white arrows indicate the growth directions. **G** Fracture of an interior vertical wall. The platy calcite becomes organized and grows from a finely granular central region (outlined) in both directions (white arrows). A thin granular layer covers the innermost surface

**Tabular calcite** is only present in the center of the interior vertical and compound walls of *Calpensia nobilis* (Fig. 3A–C). It consists of thin flat rhomboidal crystals that are tightly stacked and parallel to the inner surface (Fig. 3A). The tablets are typically spiral-shaped and the step size is the thickness of one tablet (see inset in Fig. 3B). The number of sides ranges from 4 to 6, and their diameters can reach up to 10 µm, depending on the growth stage. In a cross-sectional view, the thickness of the tablets ranges between 100 and 300 nm, and the boundaries are marked by abundant organic threads (Fig. 3C). At high magnification, the growth lines and the surface nano-roughness of the polygonal tablets are visible (Fig. 3B). Growth lines indicate that tablets spread laterally until colliding with other tablets growing at the same level (Fig. 3B).

**Irregularly platy calcite** is made up of lamellar crystals that constitute the central part of the interior vertical and compound walls in *Schizoretepora serratimargo, Schizobrachiella sanguinea* and *Pentapora fascialis*, arranged parallel to each other and to the inner surface (Fig. 3D–G). The thickness of individual lamellae is not constant because of the undulate morphology of their boundaries, and wedging out of lamellae is also frequent (Fig. 3E–G). The thickness ranges from 0.2 to 3 µm, while the lateral extensions can vary from 10 µm to several tens of micrometers. Hence the name “irregularly platy”. Slight etching and decalcification treatment on polished surfaces reveal the dense distribution of organic threads encasing the crystals (inset in Fig. 3D).

**Fig. 4:**
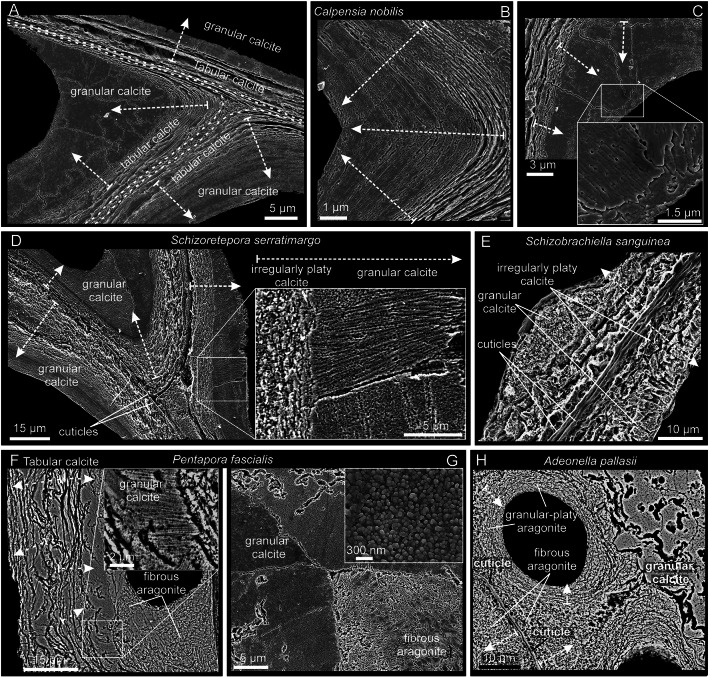
Relationship between calcitic microstructures in bimineralic bryozoans. **A**–**C** Polished sections of compound walls of *Calpensia nobilis*. **A** Tabular calcite is present from the center (dashed lines) and extends on both sides. As the wall thickens, the tablets become more and more compact until they finally grade into a thick granular calcite layer. **B**, **C** Interior walls at the corners. Granular calcite covers the innermost surface of the zooecium, becoming thicker at the corners. The grains display an internal fibrosity coincident with the marginal serrated teeth (inset in **C**). **D** Polished sections of a compound wall of *Schizoretepora serratimargo*. Similar to tabular and granular calcite, the irregularly platy calcite becomes progressively more compact until changing into granular calcite. The close-up view of the framed area demonstrates that the fibrosity is continuous across both microstructures. **E** Polished and slightly decalcified section of a thin compound wall of *Schizobrachiella sanguinea*. The transition from irregularly platy to granular calcite is barely perceptible. **F**, **G** Polished compound wall sections of *Pentapora fascialis*. As usual, the transition from irregularly platy to granular calcite is gradual. The internal fibrosity of crystals is also continuous across both layers and can be traced up to the boundary with the aragonite layer (see inset in **F**). A magnified view (inset in **G**) shows the surface nano-roughness of the granular calcite. **H** Polished compound wall section of *Adeonella pallasii.* Granular calcite is found at the interior walls of the zooecium. Fibrous aragonite spreads from the cuticle inwards and between the pores, which are in turn surrounded by granular-platy aragonite in a concentric disposition. White dashed arrows indicate the growth directions

**Fig. 5:**
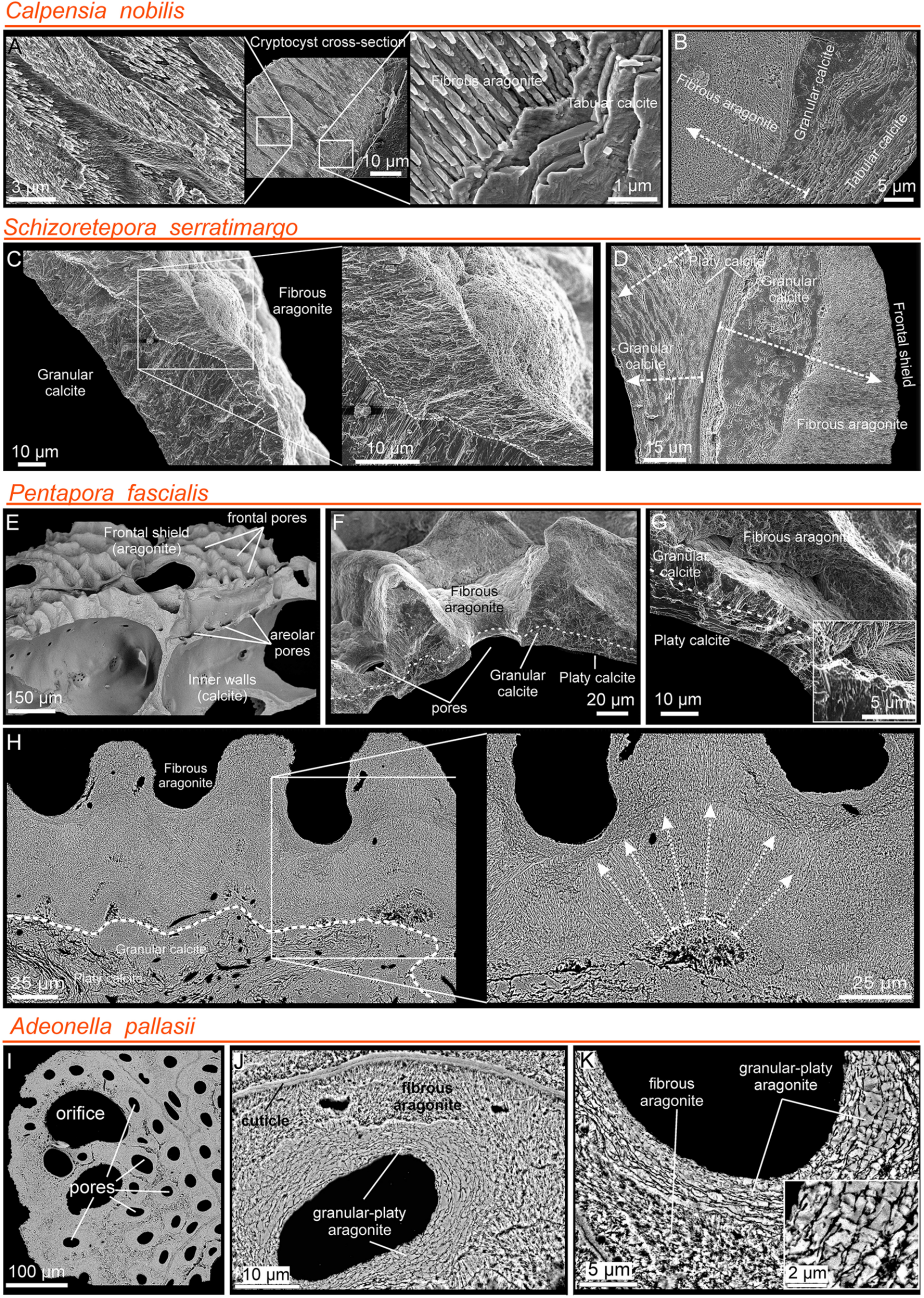
Fibrous aragonite in bimineralic bryozoans. **A** Cryptocyst of *Calpensia nobilis*. Fibrous aragonite overlying tabular calcite. **B** Cryptocyst showing the succession of layers (arrow indicates growth direction). **C** Frontal shield of *Schizoretepora serratimargo*. The granular calcite predates the fibrous aragonite. The close-up shows spherulites of aragonite, which appear nodular. **D** Frontal shield, displaying the same sequence of microstructures as in **B**. Arrows indicate growth direction.** E** Zooecia of *Pentapora fascialis* showing the main structures. **F**, **G** Frontal shield. Aragonite spherulites grow onto inner platy and middle granular calcite layers, forming protuberances around pores. The granular calcite has toothed external surfaces (inset in** G**). **H** Frontal shield showing basal layers of platy and granular calcite and outer layer of fibrous aragonite. Aragonite fibers are arranged radially (arrows in close-up). **I** Frontal shield of *Adeonella pallasii*, with numerous frontal pores. **J** Compound wall with cuticle, granular-platy aragonite surrounding the pore, and fibrous aragonite in between. **K** Granular-platy and fibrous aragonites. Platy units form angular grains (inset)

**Granular calcite** was found in all bimineralic bryozoans analyzed. It constitutes a relatively coarse layer that is always at the interior of the zooecial chambers or below the outer aragonite layers of the frontal shield (Figs. 4, 5). In cross-section, the grains range from 3 to 10 µm in width, and from less than 5 to more than 20 µm in length (Fig. 4A–H; Supplementary Fig. S5A, B), becoming particularly large in the curvature zones (i.e., at the angles) of the interior walls. They extend in the direction of growth and demonstrate irregular boundaries (pseudodendritic) with neighboring grains (Fig. 4A–C). When conveniently etched, the granular crystals display an internal fibrosity. The individual fibers are closely spaced, oriented toward the growth direction, and terminate into the small teeth of the serrated margins (Fig. 4C, D and inset in F). This internal fibrosity can also be appreciated in fractures (granular layer in Fig. 5C and inset in G). In a magnified view, the characteristic surface nano-roughness is revealed (inset in Fig. 4G). The AFM phase signal (Supplementary Fig. S5C) does not show any consistent change in composition. The size of the nanoprotrusions ranges from several tens to more than 100 nm (Supplementary Fig. S5C).

#### Aragonite microstructures

In bimineralic species, fibrous aragonite forms the outermost layer of either the cryptocyst (in *Calpensia nobilis,* Fig. 5A, B) or the frontal shield (in all other bimineralic species, i.e., *Schizoretepora serratimargo* and *Pentapora fascialis*, Fig. 5C–H), which is secreted onto a basal calcitic layer (tabular, platy, or granular) (Fig. 5B, D). Fibrous aragonite is made of thin and long crystals arranged into spherulites (Fig. 5C, F, H), whose growth surfaces protrude toward the frontal surface, giving it a nodular aspect (Fig. 5C). This nodularity is enhanced when the frontal shield is perforated by the frontal or areolar pores (Fig. 5E, F). The size of the fibers of the cryptocyst of *C. nobilis* is smaller (60–150 nm in width, 2–4 µm in length, Fig. 5A) than those of the frontal shields of the ascophoran species. For instance, in *P. fascialis*, the fibers range from 200 to 600 nm in width and from 2.5 to > 10 µm in length (Supplementary Fig. S6A–C). The nano-roughness of the surface can be seen at higher magnification (Supplementary Fig. S6B, C) but no contrast differences were detected in the AFM phase signal (Supplementary Fig. S6D). The size of the nanoprotrusions may vary from several tens to over 150 nm, slightly larger than those analyzed in the granular calcite (see Supplementary Fig. S6C).

Granular-platy aragonite was only found in *Adeonella pallasii*, surrounding the pores of the frontal shield (Figs. 4H, 5I–K; Supplementary Fig. S2M-O). In cross-section, irregularly platy aragonite units can be confused with those of the irregularly platy calcite. Nevertheless, the platy aspect is less well developed and the crystals often appear subdivided transversely, leading to the formation of granular units with angular outlines (Fig. 5I-K), sometimes reminiscent of crystal faces (Fig. 5K, inset). The sizes of grains vary from less than 1 µm to more than 5 µm in length and from less than 200 nm to 1 µm in width.

#### Calcitic bryozoans

**Fig. 6:**
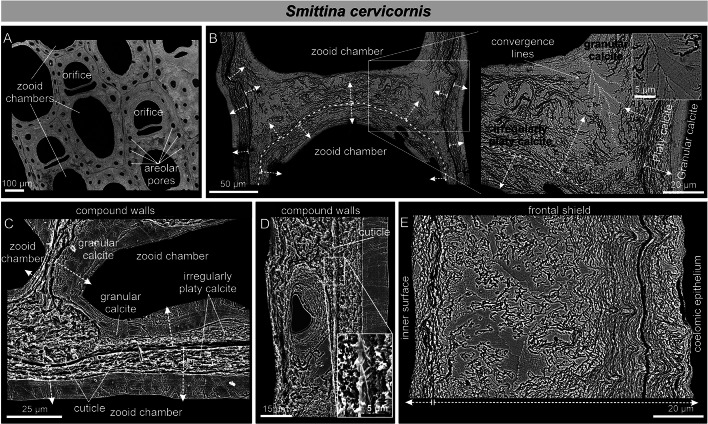
SEM images of the irregularly platy and granular calcites of *Smittina cervicornis.* **A** Polished colony fragment. The section plane cuts the zooids longitudinally. **B** Polished surface of the compound walls between adjacent zooids. The white dashed arrows indicate the growth direction from the cuticle between the zooids (marked with broken lines) toward the interiors of the chambers (detailed view in the inset). The close-up of the framed area shows the transition from platy to granular calcite. Due to the high curvature of the wall, the grains (growing at high angles to the wall) converge along irregular bisecting lines (thin broken lines). **C**, **D** Polished-etched compound wall in longitudinal section. The growth direction proceeds from the cuticle toward the zooid interior, with the irregularly platy calcite forming first, followed by the granular calcite. The irregularly platy calcite surrounds the areolar pores and their laminae display a concentric disposition (e.g. **D**). The fibrous nature of the cuticle can be appreciated (inset in **D**). **E** Polished cross-section of a frontal shield. Two growth directions are discernible: (1) toward the zooid interior (left) and (2) toward the frontal surface (bottom dashed arrows)

The only bryozoan analyzed that possessed a wholly calcitic skeleton was *Smittina cervicornis* (Fig. 6; Supplementary S3A–C). The microstructures that are secreted are irregularly platy and granular calcite (Fig. 6A, B), identical to those found in bimineralic bryozoans. The layer adjacent to the cuticle in compound walls is made of irregularly platy calcite (Fig. 6C, D), while a granular calcite layer covers the inner surfaces of the zooecium. As the frontal shield thickens, there is a smooth transition from a platy to a granular microstructure (of the kind described in bimineralic bryozoans). Toward the surface, the irregularly platy calcite becomes more undulated, and the growth increments become more marked (Fig. 6E). Fibers reveal a surface nano-roughness under high magnification (Supplementary Fig. S7A). The phase signal shows changes in contrast, indicating variations in composition. Nanoprotrusions range from a few tens to more than 100 nm (see Supplementary Fig. S7B, C), similar to those in the granular calcite of bimineralic bryozoans (Supplementary Fig. S5B).

#### Aragonitic bryozoans

**Fig. 7:**
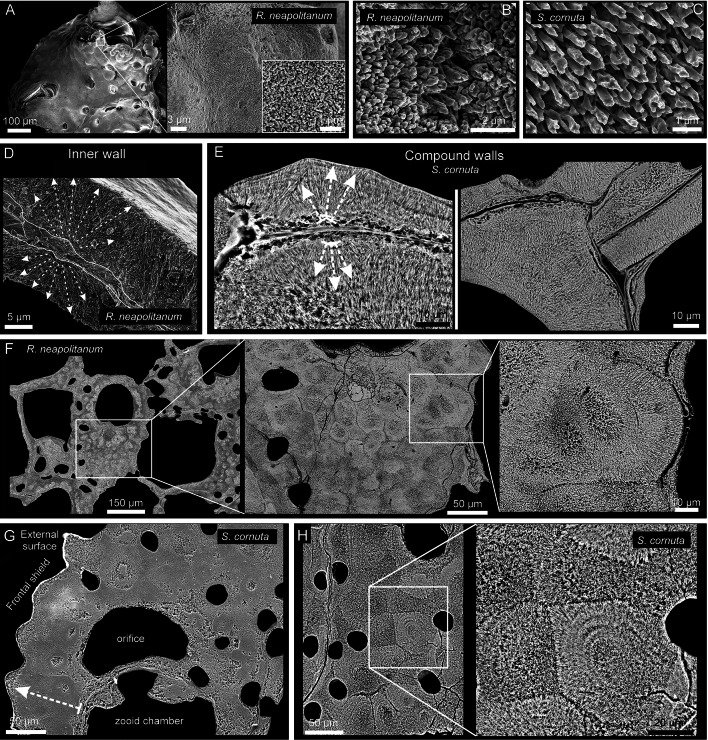
SEM images of the fibrous aragonite of *Rhynchozoon neapolitanum* (**A**, **B**, **D**, **F**) and *Schizomavella cornuta* (**C**, **E**, **G**, **H**). **A** Autozooid and adventitious avicularia of *R. neapolitanum*. The close-up view to the right shows the nodular reliefs. The small inset shows the granular aspect of the external surface. **B**, **C** Detailed views of the bundles of aragonite fibers of both species. **D** Fracture of an interior wall of *R. neapolitanum*. The aragonite crystals grow bidirectionally from a granular center, resulting in a sandwich structure. **E** Polished compound wall sections of *S. cornuta*. The aragonite crystals grow from the cuticle toward the zooid interiors in both directions. **F** Polished fragment of *R. neapolitanum*. The section plane cuts through the frontal shield, showing the outlines of the spherulites. **G** Polished section of a zooid of *S. cornuta*. The spherulites produce a nodular appearance of the frontal shield. **H** Polished section along the frontal shield. The close-up of the framed area shows the outlines of the spherulites composed of radiating fibers and displays marked growth increments. White dashed arrows in **D**, **E** and **G** indicate growth direction

*Rhynchozoon neapolitanum* and *Schizomavella cornuta* (Fig. 7) are the only species whose skeletal structures (interior walls, compound walls, and frontal shields; Supplementary Fig. S4) are made entirely of fibrous aragonite. As described above for bimineralic bryozoans, fibrous aragonite consists of fibrillar crystals arranged into spherulites. The convex, free surfaces of the spherulites produce a nodular aspect to the outer surface of the frontal shield (Fig. 7A). Individual fibers are 2 to ~ 40 µm long, depending on the size of the spherulite, and 0.5–1 µm wide (Fig. 7B, C, E, F). In the interior walls, the fibers nucleate onto an unstructured, granular central layer and spread on both sides toward the inner surfaces. The result is a wall with a sandwiched structure (Fig. 7D). In the compound walls that separate adjacent zooids, the fibers nucleate from the cuticle (placed at the center) toward the interior of the zooecium (Fig. 7E). In both cases, the inner surfaces are relatively smooth. At the frontal shield, the aragonite fibers spread toward the exterior (Fig. 7F–H). During the thickening of the frontal shield, the spherulites merge with each other. In polished sections, nucleation centers are clearly visible (Fig. 7F). The growth surface of the spherulites is convex or straight, depending on whether it grows free or abuts a neighboring spherulite (Fig. 7G, H).

### Crystallography

**Fig. 8:**
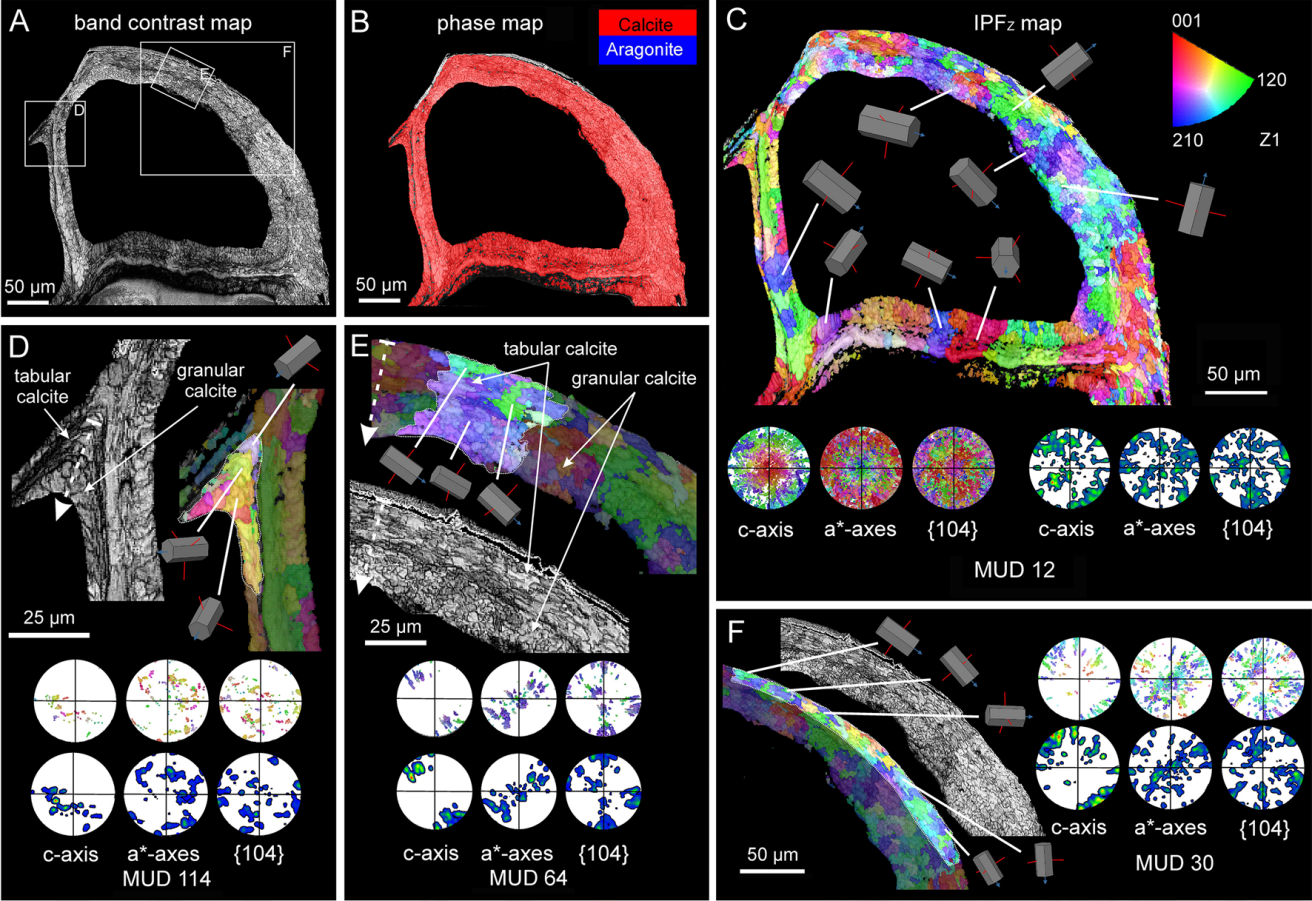
EBSD analysis of the interior walls of a zooid of *Calpensia nobilis*, comprising tabular and granular calcite. **A** Band contrast map. The lighter or darker regions correspond to stronger or weaker EBSD diffraction signals, respectively. The framed areas are analyzed in detail in** D**, **E** and **F**. **B** Phase map showing that all the interior walls are made of calcite. **C** EBSD map. The color triangle is the orientation color key. The varied colors indicate a non-preferred orientation of the crystallographic axes, as shown by the pole figures. The low MUD for the entire map (12) is partly due to the changing orientation of the wall. The orientation of selected grains is also provided by the unit cells. **D** Detailed view of an interior wall corner. The tabular calcite grades into granular calcite without a significant change in orientation (note similar colors and unit cell orientations). The pole figures indicate that the c-axes are at a high angle to the section plane. **E** Magnified area of the wall cross-section. The selected subset comprises both tabular and granular calcite. No changes in the orientation from one microstructure to the other are observed (as indicated by the unit cells). The pole figures indicate that the c-axes are roughly parallel to the section plane and to the elongation of the wall. **F** Subset of the tabular calcite layer only. The c-axes lie parallel to the section plane and to the growth surface, showing a change in orientation coinciding with the curvature of the wall (see the unit cells). Color key to the top right of **C**, valid for all maps

The crystallography of the tabular and granular calcite of *Calpensia nobilis* is shown in Fig. 8. The selected map was done on the interior walls of a zooid chamber. The boundary between the tabular and granular (on the inner side) microstructures is delineated in the band contrast map (Fig. 8A), demonstrating good signal strength of the measurement. The phase map indicates that the interior walls are made entirely of calcite (Fig. 8B). The entire EBSD color orientation map (IPFz map, Fig. 8C) reveals a broad color variation, which suggests no preferred orientation of the calcite crystals. The pole figures of the map display a scattered distribution of the crystallographic axes (Fig. 8C), due to the curvature of the walls. The MUD value (12) for the map indicates a low co-orientation of the crystallographic axes. Locally, similar colors extend from the tabular to the granular microstructures, delineating relatively big “crystallographic domains” with a uniform orientation. This indicates crystallographic continuity between the two microstructures. This continuity can be better appreciated in the selected subsets of Fig. 8D and E (MUD values 114 and 64, respectively). If only the tabular calcite layer is selected, the c-axes are roughly parallel to the growth surface (Fig. 8F). The maxima spread in the contour pole figures (with a MUD value of 30), partly due to the c-axes following the curvature of the wall (as indicated by the unit cells, Fig. 8F).

**Fig. 9:**
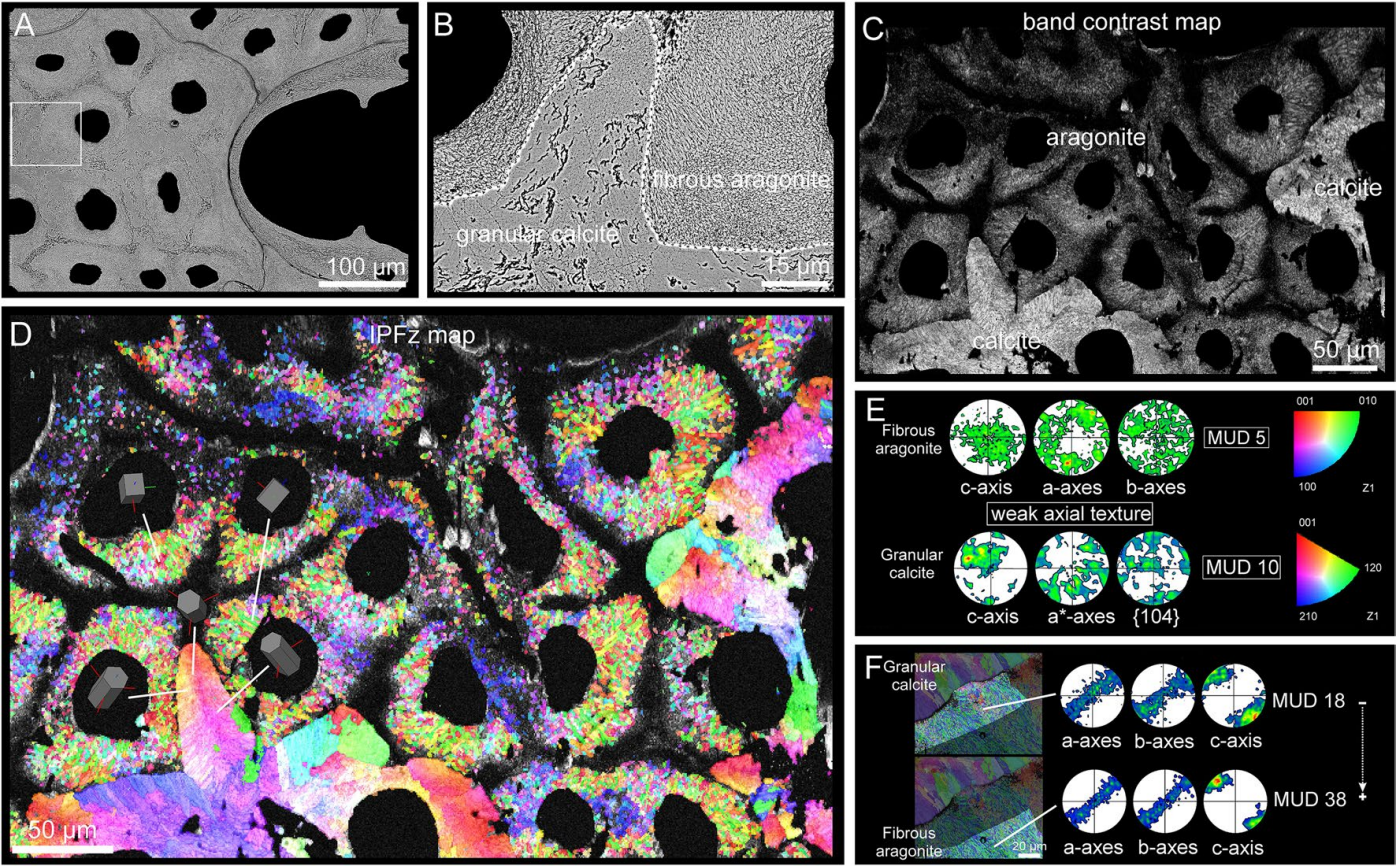
EBSD analysis of the granular calcite and the fibrous aragonite of *Pentapora fascialis*. **A**, **B** SEM images of a region similar to that analyzed for EBSD, corresponding to a longitudinal cross-section of the frontal shield. The middle layer of granular calcite (see Fig. 5E–G) is visible between the fibrous aragonite that surrounds the pores. The boundary between the calcite and the aragonite microstructures is visible (white dashed line). **C** Band contrast map. The calcite shows stronger EBSD diffraction signals (lighter colors than the aragonite). **D** IPFz map. The broad variety of colors indicates widely scattered orientations of the crystallographic axes for both microstructures. Orientation color keys are provided in **E**. **E** Contour pole figures for the fibrous aragonite (top) and the granular calcite (bottom). The crystallographic axes depict a weak axial texture, with the c-axis as fiber axis. Accordingly, the MUD values are very low. The triangles are orientation color keys for aragonite and calcite. **F** Selected subsets for the fibrous aragonite at two different growth stages. The selected area to the top comprises an initial growth stage starting at the boundary with the granular calcite. The region to the bottom belongs to a more advanced growth stage. The contour pole figures and their MUD values (18 and 38) indicate that the texture becomes stronger with growth

The crystallography of the granular calcite and the fibrous aragonite microstructures of bimineralic bryozoans is represented by the *Pentapora fascialis* example (Fig. 9A, B). The selected map was obtained in a cross-section that cuts obliquely through the frontal shield. In the band contrast map (Fig. 9C), the regions corresponding to the fibrous aragonite (composed of smaller crystals) present a lower signal strength (darker areas) than those of the granular calcite (lighter areas). The phase map (Supplementary Fig. S2J-L) supports the distribution of aragonite surrounding the pores in the outermost layer of the frontal shield (blue color, Supplementary Fig. S2L). The granular calcite (red color, Supplementary Fig. S2L) is progressively exposed between the aragonite as the oblique sectioning plane reaches the basal calcitic layers of the frontal shield (see distribution of layers in Fig. 5E–H). An overview of the entire IPFz map (Fig. 9D) reveals a wide color variation for both microstructures, suggesting that the crystals are inconsistently oriented. The contoured versions of the pole figures of the calcitic and aragonitic microstructures show weak axial textures in both cases (Fig. 9E), with scattered maxima for the c-axes, indicating an orientation roughly perpendicular (aragonite) or oblique (calcite) to the section plane. The low MUD values (10 and 5 for calcite and aragonite, respectively) are evidence of the poor texture (Fig. 9E). An analysis of the change in co-orientation of the microstructures with growth is shown in Fig. 9F. We have cropped out areas corresponding to two different developmental stages of the fibrous aragonite. According to the lesser spread of the pole maxima of the contoured pole figures, and to the increase of the MUD values (from 18 to 38), the fibers become more co-oriented with growth.

**Fig. 10:**
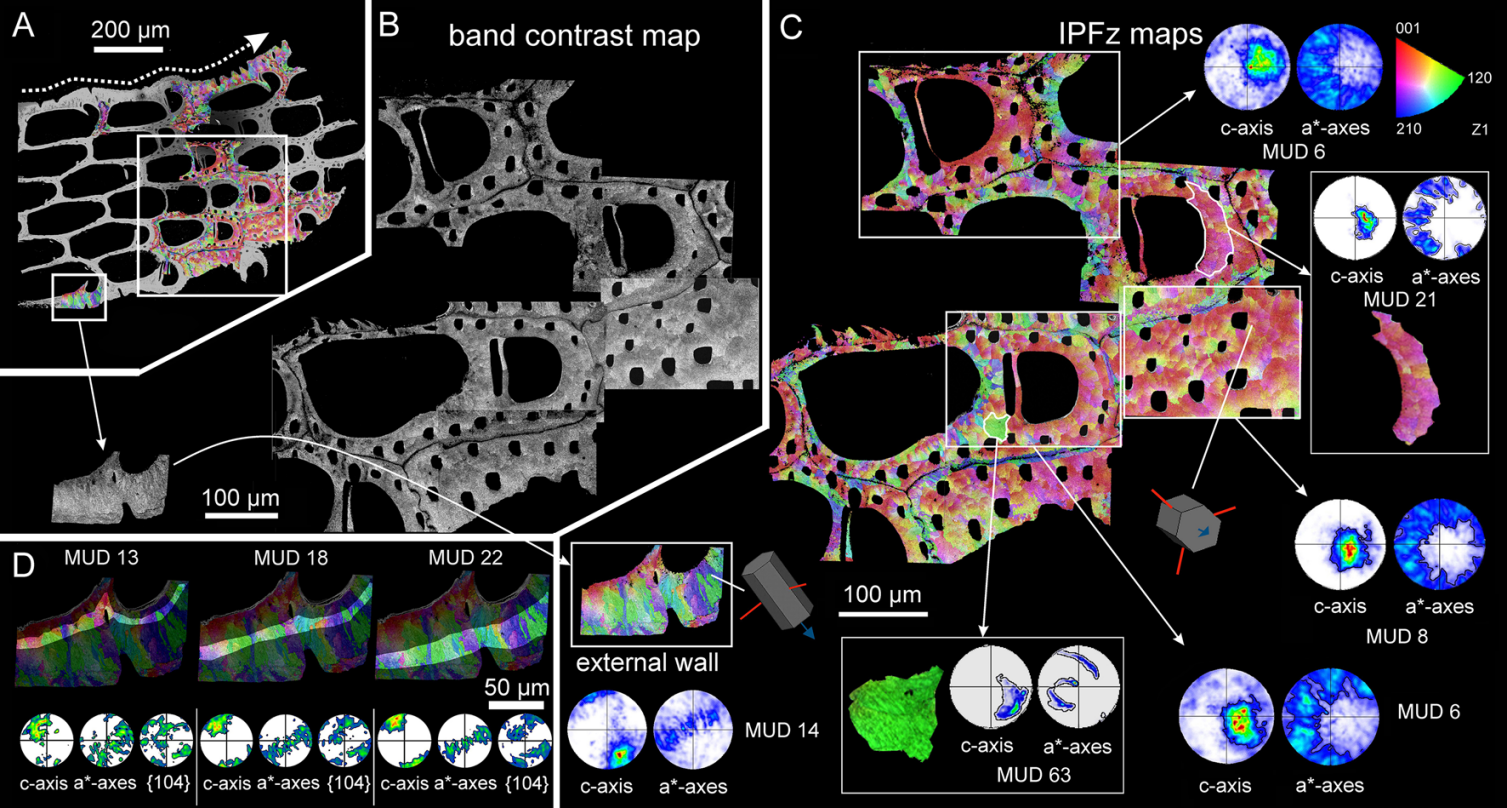
EBSD analysis of the platy and granular calcites of *Smittina cervicornis.* **A** SEM image of a longitudinal cross-section of a colony fragment. The section plane cuts throughout different zooid chambers and covers the entire width of the colony. The dashed wavy arrow indicates the colony growth direction.** B** Band contrast maps of the areas framed in **A**, showing a good diffraction signal strength for all the measurements. **C** EBSD orientation color maps (IPFz map) for the areas in B and contoured pole figures for framed regions and subsets (delineated with white lines). The MUD values are low (between 6 and 14) for the framed areas. The reddish colors predominate (i.e., the c-axis is at a high angle to the section plane), except in areas adjacent to the cuticle between zooids (compound walls) and at the external walls, where the c-axes are roughly parallel to the sectioned surface (subset to the bottom left). The calcite crystals are organized into big crystallographic domains with relatively high internal misorientations (bottom center selected green subset, with an MUD value of 63). The color triangle is the orientation color key. **D** Selected areas along the external wall at increasing growth stages (from left to right). The lesser spread of the maxima in the contour pole figures and the increasing MUD values (from 13 to 22) indicate that the texture becomes stronger with growth

The crystallography of the irregularly platy and granular calcites of *Smittina cervicornis* is shown in Fig. 10. The analysis was performed on a large area comprising a longitudinal cross-section across several zooid chambers (Fig. 10A, B). The bulk of the colony is made of calcite (see the phase map in Supplementary Fig. S3). The IPFz map (Fig. 10C) reveals a high incidence of reddish colors, which indicates that the c-axes of calcite are at a high angle to the sectioning plane. This is also shown by the contoured pole figures of Fig. 10C. The exceptions are the external walls and the areas adjacent to the cuticles (in green and blue), where the c-axes lie at a low angle to the surface (subset to the bottom left in Fig. 10C). The crystallographic domains extend to the platy and granular calcites, which indicates no distinction in crystallographic orientation. This is shown by the slight color variation and the slight dispersion of the pole figure maxima of the delineated and zoomed areas in Fig. 10C (with MUD values of 21 and 63). All the pole figures in Fig. 10C display an axial texture, with the c-axis as the fiber axis. Similar to the fibrous aragonite, the granular and irregularly platy calcites become more co-oriented with growth, as corroborated by the lesser spread of the pole maxima and the increase of the MUD values of areas corresponding to the different growth stages (Fig. 10D).

**Fig. 11:**
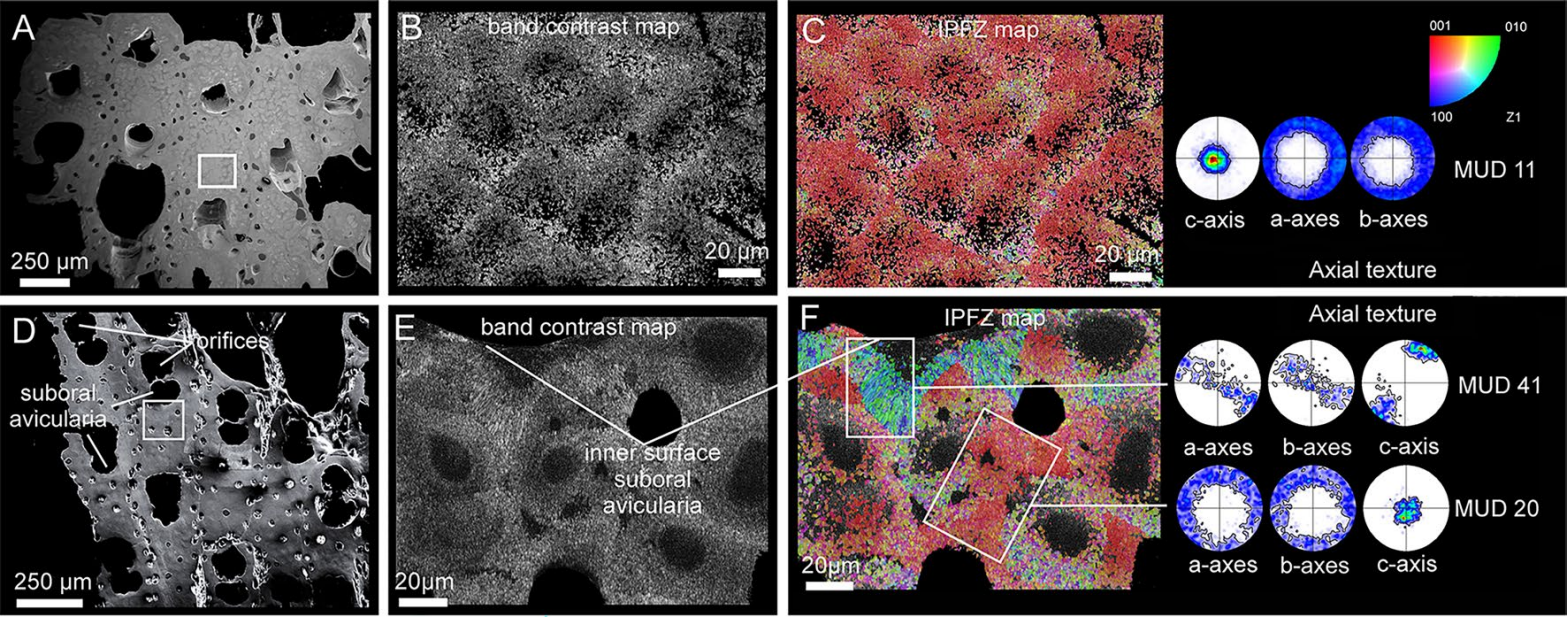
EBSD analysis of the fibrous aragonite of *Rhynchozoon neapolitanum* (**A**–**C**) and *Schizomavella cornuta* (**D**–**F**)*.* **A**, **D** SEM images of the polished colony fragments. The section planes cut across the frontal shields of the zooids. The framed areas correspond to the regions analyzed with EBSD. **B**, **E** Band contrast maps showing darker hues at the centers of the spherulites, where the signal strength of the measurement is weak or absent. **C**, **F** IPFz maps and pole figures (contoured version), displaying clear axial textures. The c-axes are perpendicular to the section plane, with the exception of the region around the sub-oral avicularia orifice in *S. cornuta* (**F**, top framed area), where the c-axes of the fibers are oriented roughly parallel to the section plane

The crystallography of the predominantly aragonitic bryozoans is represented by the examples of *Rhynchozoon neapolitanum* (Fig. 11A–C) and *Schizomavella cornuta* (Fig. 11D–F). The areas for EBSD measurements were selected on a longitudinal cross-section of the frontal shields (Fig. 11A, D). EBSD phase maps indicate the aragonitic nature of the samples (Supplementary Fig. S4). The spherulites are discernible in the band contrast maps (Fig. 11B, E; compare to Fig. 7E, G); their centers have a weaker signal strength (darker areas) because the fibers are thinner and disposed at a high angle to the section plane. The IPFz maps (Fig. 11C, F) show that the fibers are oriented predominantly perpendicular to the section plane (red color). Exceptions were found in *S. cornuta,* where the c-axes of the fibers are locally parallel to the section plane and perpendicular to the inner surface of the orifice of the sub-oral avicularia (top framed area in Fig. 11F). The pole figures display a neat axial texture for both samples, with the c-axis as the fiber axis. The MUD values indicate a weak-to-medium texture.

## Discussion

### General structure and mineralogy of cheilostome bryozoans

Cheilostomes develop a complex colony structure with a widespread and high level of zooid polymorphism (Figs. 1, 2). In addition, feeding autozooids, defensive avicularia, brooding chambers (ovicells and gonozooids), and structural kenozooids are often found (Schack et al. 2019; Taylor 2020). Considering the larger variety of colony forms and functional zooids, and the higher colony growth rates of cheilostomes (due to their better ability to obtain food) compared to cyclostomes, it is not surprising that they have become the most successful bryozoan group from the Late Cretaceous to date (McKinney 1992; Schack et al. 2019; Taylor 2020; Taylor and Waeschenbach 2015).

The organic fraction present in the zooidal skeleton consists of the outer cuticle and the inter- and intra-mineral organic matter. The first studies performed on histological preparations were done with periodic acid Schiff and Mallory triple stain tests and revealed a cuticle composition predominantly of mucopolysaccharides, proteins, and chitin (Tavener-Smith and Williams 1972). Lombardi et al. (2023) demonstrated a similar composition in the intra-mineral soluble organic matter of three Antarctic calcitic bryozoans’ skeletons.

Our samples included calcitic, aragonitic, and bimineralic bryozoans (Supplementary Figs. S2-S4). In all bimineralic species, aragonite was always found in the frontal shield (or cryptocyst in *Calpensia nobilis*), while calcite constituted the structure of the interior compound walls and the basal layers of the frontal shield. According to numerous studies summarized by Taylor et al. (2009), there is a strong latitudinal pattern related to the mineralogy of the gymnolaemate bryozoans, since the number of aragonitic and bimineralic species increases toward the equator. This pattern was explained by the different solubilities of the calcium carbonate mineral phases, as high-Mg calcite and aragonite are more vulnerable at high-latitude cold waters. Hence, solubility seems an important factor limiting the geographical extent of species according to their mineralogy (Kuklinski and Taylor 2009). This latitudinal pattern is consistent with the mineralogy described in our samples, which were collected in the Adriatic Sea (see Materials and methods).

### Relationships between microstructures

In the eight species of cheilostome bryozoans analyzed in this study, we detected five microstructures: three calcitic (tabular, irregularly platy, and granular) and two aragonitic (fibrous and granular-platy). To determine the growth sequences, we used the following criteria: (1) the cuticle is always the first structure to be secreted (Tavener-Smith and Williams 1972); (2) growth fronts can be deduced from the distribution and shape of crystal growth lines (Figs. 5H, 6C–E, 7E, F, H); (3) the contours of platy calcite units are considered as growth surfaces when they are subparallel to each other and, eventually, to the growth lines of granular calcite (Fig. 6E); (4) the grains on both sides of the boundaries between converging growth fronts of the granular calcite point in the growth direction (Fig. 6B, inset); (5) the spherulites spread from the nucleation center outwards in all directions (Fig. 7F–H).

**Fig. 12:**
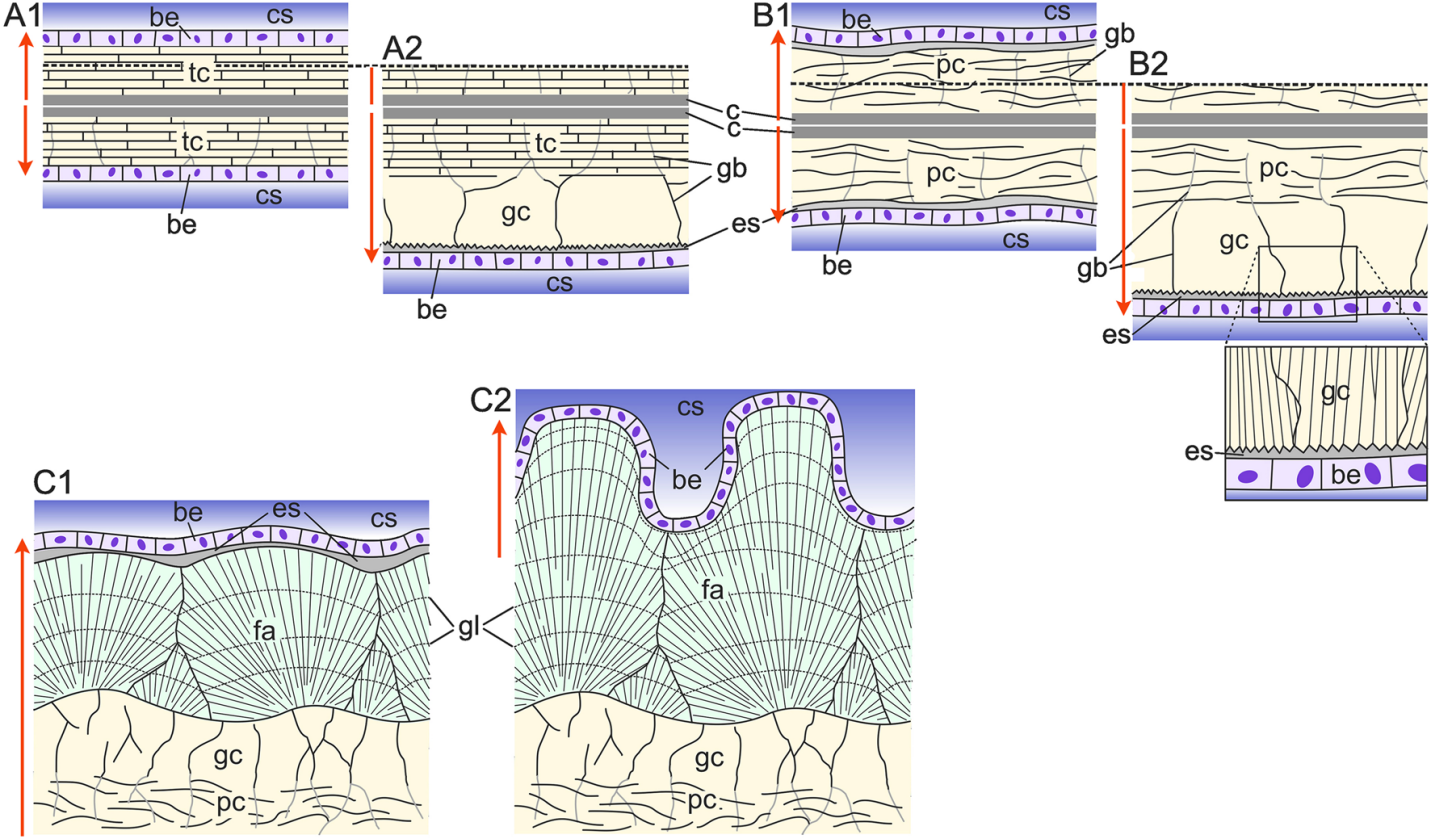
Growth sequences of microstructures and relation with the secreting epithelium in the compound walls (**A**, **B**) and frontal shield (**C**). **A** Compound walls of *Calpensia nobilis*. Walls grow from the cuticles in both directions toward the zooecium interiors. During the secretion of the tabular calcite (A1), the biomineralizing epithelium is in close contact with the shell growth surface, although there is always a small, almost negligible, extrapallial space. During subsequent secretion of the granular calcite (A2), there is a wide intervening extrapallial space. **B** Compound walls of the rest of either calcitic or bimineralic species. The walls are made by a cuticle followed by irregular platy calcite (B1) and granular calcite (B2). The biomineralizing epithelium is at a certain distance from the shell growth surface. The zoom up in B2 shows the relationship between the biomineralizing epithelium and the serrated shell growth surface in more detail. **C** Formation of the frontal shield in bimineralic species (e.g., *Pentapora fascialis*). It is formed by superposed layers of irregular platy calcite, granular calcite, and spherulites of fibrous aragonite. During active secretion (C1), the soft body is separated from the shell growth surface by a wide intervening extrapallial space. It is a smooth replicate of the shell surface topography. Secretion ceases first at the depressions and continues in the elevations, as indicated by growth lines (compare to Fig. 5H). When secretion of the frontal shield comes to an end, the biomineralizing epithelium adheres to the shell surface and becomes inactive (C2). Arrows indicate the growth directions. In A2 and B2, only the growth of one of the walls is shown (the broken lines are intended as references). be: biomineralizing epithelium; c: cuticle; cs: coelomic space; es: extrapallial space; fa: fibrous aragonite; gb: grain boundary; gc: granular calcite; gl: growth line; pc: irregularly platy calcite; tc: tabular calcite

We observed recurrent relationships between the calcitic microstructures of both bimineralic and calcitic cheilostomes. Tabular calcite is the first microstructure secreted in *Calpensia nobilis*, and it grows from the cuticle (at the center of the compound walls) toward the zooecium interior. As the wall thickens, the tablets, which are stacked parallel to each other and surrounded by organics threads (Fig. 3C), gradually shift to a granular layer that ultimately covers the inner surfaces of the zooecium (Figs. 4A–C (white dashed arrows), 12A). The same applies to the irregularly platy calcite. It is the first layer secreted at the center of the compound walls in the bimineralic bryozoans *Schizobrachiella sanguinea*, *Schizoretepora serratimargo*, and *Pentapora fascialis* (Figs. 3D–F, 4D–F) and in the calcitic bryozoan *Smittina cervicornis* (Fig. 6). The irregularly platy units grow from the cuticles in opposite directions toward the interiors of both zooids. Similar to the tabular calcite, the irregularly platy calcite gradually changes into granular calcite with growth (see white dashed arrows in Figs. 3D–F, 4D–F, 6B–D, 12B).

The crystallographic continuity observed between either the tabular or the irregularly platy calcite and the granular calcite (Figs. 8C–E, 10C) indicates that the microstructural change is merely due to the disappearance of the organic membranes typical of the former two microstructures. The only explanation we envisage is that, with wall thickening, there is a progressive decrease in the availability of organic components in the solidifying medium (extracellular space). The result is the formation of large calcite grains devoid of occluded organic membranes that are typical of the granular layer. Schoeppler et al. (2019) explained the transition from aragonitic granular to columnar to nacreous layers in different mollusc shells through a process analogous to that of directional solidification, well known in the field of metallic materials science (Schoeppler et al. 2018). This process in mollusc shells is unlikely to be influenced by shifts in temperature, because mineralization takes place at ambient temperature. However, supersaturation, the mineral deposition rate and the amount of organic matter have to be considered. Supersaturation would be reflected in the number of crystals (high: many crystals; low: few crystals), which is not observed in the walls of cheilostomes. Accordingly, the transition between microstructures may take place through a reduction in either the growth rate or the amount of available organic matter. At faster growth rates, the organic molecules would tend to be entrapped within the crystals, whereas at slower growth rates, they would be repelled and attach to the growth fronts, eventually forming organic membranes. Although we cannot rule out either possibility, there is neither evidence nor a clear reason why the rate of calcification should decrease during skeletal wall thickening. We opt for the possibility that a decrease in the number of organic biomolecules released by the mineralizing tissue of the animal over time is responsible for the observed microstructural changes, without affecting the crystallographic continuity. A feasible proxy for growth rate is the Mg content of the calcite crystals (Gabitov et al. 2014; Nielsen et al. 2016). However, this analysis was not carried out in this study.

In the primarily aragonitic cheilostomes *Rhynchozoon neapolitanum*, and *Schizomavella cornuta*, the bulk of the colony is made uniquely of fibrous aragonite (Fig. 7). However, no microstructural variations were observed in the different walls, where the fibers were always organized into spherulites, displaying a strong axial texture (Fig. 11). The same invariance was noted in the fibrous aragonite that forms the frontal shields (or cryptocyst in *C. nobilis*) of the bimineralic cheilostomes studied (Fig. 5A–H).

Regarding the granular-platy aragonite found around the pores in *Adeonella pallasii* (Figs. 4H, 5I–K), we observed that the irregularly platy units are often subdivided into granular crystals with angular edges. Similar to the irregularly platy calcite, the platy appearance of aragonite may be due to the intercalation of organic membranes. Despite the lack of crystallographic data on these microstructures, it is possible that both granular and irregularly platy aragonite are crystallographically continuous.

### The relation of the growing crystals with the secretory epithelium

Among the microstructures described in our study, only the tabular calcite of *Calpensia nobilis* looks relatively sophisticated, i.e., far from the morphologies of abiogenic calcites (Fig. 3A–C). It is a material made of well-defined, parallel, flat lamellae. This suggests the existence of a closely adjacent secretory epithelium that levels off the surfaces of the growing crystals (Fig. 12A1). Conversely, the other microstructures (irregularly platy calcite, granular calcite, fibrous aragonite, and granular-platy aragonite) present a characteristic inorganic-like appearance, and evidence that the epithelium is at a distance from the forming crystals (Fig. 12A2, B, C1).

The lamellae of the irregularly platy calcite display uneven boundaries marked by organic membranes. In the likely case that individual organic membranes form at once (i.e., they are parallel or subparallel to growth lines; Figs. 3F, 6E), their sinuous contours and the great variation in shape and extension imply some degree of freedom of the growth surface. This is understandable if there is not a tight connection with an even secretory epithelium surface (Fig. 12B). Otherwise, the lamellae would tend to be flat, regular in size, and well ordered parallel to the growth surface (as in the tabular calcite of *C. nobilis*; Fig. 12A1). Regarding the granular calcite, the grains show an internal fibrous substructure. Each fiber coincides with a tooth of the serrated growth surface. Clearly, teeth are sectioned small rhombohedra (Fig. 4C, D, F). Thus, the surfaces of grains are comparable to micro-sized dog-tooth calcite (Fig. 5G, inset). This suggests that the growth fronts of grains are at a distance, even if small, from the epithelium (Fig. 12A2, B2).

The fibrous aragonite microstructure is always made by spherulites (see Figs. 5, 7) that extend until colliding with their neighbors and then grow on top of each other. They give the external surfaces a nodular aspect (Figs. 5C, E, 7A), which is evidence that their growth surfaces are free to bulge, out of the reach of an epithelium leveling them off (Fig. 12C1). The distribution of growth lines (Fig. 5H) indicates that, when the frontal shield is about to be complete, growth ceases at the depressions, but continues at the elevations. This indicates that the biomineralizing epithelium ceases to secrete at the depressions, while it is actively secreting at the elevations. When the frontal shield formation is complete, the epithelium adheres all along its surface and becomes inactive (Fig. 12C2).

All the characteristics of the aragonitic and calcitic microstructures of cheilostome bryozoans mentioned above suggest an origin via remote biomineralization, i.e., the process which occurs when the secretory epithelium is not in close contact with the forming crystals (Checa 2000; Chinzei and Seilacher 1993). This is unlike other groups, e.g., bivalves and brachiopods, in which the thicknesses of the extrapallial spaces (i.e., the distance between the cells and the forming crystals) are on the order of 100 nm (Checa et al. 2014) and 50 nm (Simonet Roda et al. 2019), respectively. In stenolaemate bryozoans, the distance increases to 150–200 nm (Nielsen and Pedersen 1979). We cannot give an estimate of the thickness of the extrapallial space in gymnolaemate bryozoans, but it must be well above those dimensions.

### Growth by space competition

Space competition happens in crystal aggregates with a common growth front. The crystals that grow with their fastest growth axes at a high angle to the growth front survive and become larger at the expense of those growing more obliquely, which tend to be extinguished (Grigor’ev 1965; Rodríguez-Navarro and García-Ruiz 2000). Consequently, with time, the spread of crystallographic axes lessens. This applies to a wide variety of molluscan microstructures (Crippa et al. 2020; Stevens et al. 2017; Ubukata 1994), and the chicken eggshell (Hincke et al. 2012).

With EBSD, we supported an increasing crystallographic ordering at subsequent development stages of the irregularly platy-granular calcite association (Fig. 10D) and the fibrous aragonite microstructures (Fig. 9F). This is consistent with a growth process of competition for space and is the first case so far reported in bryozoans.

## Final remarks

With the exception of tabular calcite, the microstructures present in cheilostomes are much more abiogenic-like compared to those of cyclostomes, which produce tabular and foliated calcite (Grenier et al. 2023; Taylor and Weedon 2000), or to those of other organisms such as brachiopods (tabular, fibrous calcite) (Williams 1970) and molluscs (e.g., foliated, nacre, crossed-lamellar) (Bøggild 1930). In all of these instances, the thickness of the extrapallial space is negligible and there is strict secretory control by an adjoining epithelium (see Checa 2018 for molluscs, and Simonet Roda et al. 2019 for brachiopods). As commented on above, this is not the case in cheilostome bryozoans, where remote biomineralization seems widespread. The question remains, however, whether remote mineralization is favorable in terms of, e.g., a lower metabolic cost (Lowenstam 1981; Lowenstam and Weiner 1989; Mann 1983; Palmer 1983, 1992). Sophisticated microstructures are, in general, highly functional materials and their biomechanical performance is particularly good (e.g., Currey 1977; Currey and Taylor 1974). In this sense, it is possible that cheilostomes make a low investment in high-performance materials, which was not an obstacle to their extraordinary evolutionary success.

## Conclusions

1. We have identified five basic microstructures in cheilostome bryozoans: Tabular, irregularly platy and granular calcite, as well as fibrous and granular-platy aragonite.
2. The three calcitic microstructures form recurrent associations. The SEM and EBSD results indicate that both tabular and irregularly platy calcite are structurally continuous into granular calcite. The differences can be attributed to a significant reduction of the observed organic matrix.
3. Both SEM and EBSD data allowed us to reconstruct the growth directions of the microstructures, filling in the interior walls and frontal shields (or cryptocysts). Depending on the species, either tabular or irregularly platy calcite is secreted first and later transformed into granular calcite. In bimineralic cheilostomes, fibrous aragonite is always the last microstructure to be secreted.
4. The granular-platy microstructure was only found in *A. pallasii*, around the pores of the frontal shield.
5. The increase in the MUD values (derived from the contoured pole figures) for both the calcitic and aragonitic microstructures suggests competitive growth, which is described for the first time in cheilostome bryozoans.
6. Apart from tabular calcite, the rest of the microstructures show evidence that the epithelium is not in direct contact with the surfaces of the growing crystals. This demonstrates a case of remote growth, which seems widespread in cheilostome bryozoans but is uncommon among invertebrates.

### Supplementary Information

Below is the link to the electronic supplementary material.

Supplementary file1 (PDF 4236 KB)

## Data Availability

The data that support the findings of this study are included in this study (and its supplementary information). This and additional sets generated during and/or analyzed during the current study are available from the corresponding author upon reasonable request.

## Declarations

### Material

Specimens of the eight cheilostome species studied were collected with the help of Maja Novosel (University of Zagreb) and Javier Souto (University of Vienna). Xiaofei Yin (Ludwig-Maximilians Universitat, Munich) helped in sample preparation and EBSD data evaluation.

### Programs used list

Oxford AZtec and CHANNEL 5 HKL for EBSD data acquisition and analysis. ORS Dragonfly Pro^TM^ for Micro-CT image processing. Park XEI for AFM image processing and analysis. CorelDRAW 2020 for the composition of the figures.
