# Supplementary material for: Skeletal microstructures of cheilostome bryozoans (phylum Bryozoa, class Gymnolaemata): crystallography and secretion patterns
Source: Mar Life Sci Technol. 2024 Jun 7;6(3):405–24. doi: 10.1007/s42995-024-00233-1 (PMC11358562; doi:10.1007/s42995-024-00233-1)
Supplement: Supplementary file 1 — Supplementary file1 (PDF 4236 KB) [file 42995_2024_233_MOESM1_ESM.pdf]

Skeletal microstructures of cheilostome bryozoans (phylum Bryozoa, class Gymnolaemata): crystallography and secretion patterns

Christian Grenier[1], Erika Griesshaber[3], Wolfgang Schmahl[3], Björn Berning[4], Antonio G. Checa[1,2]*

[1]Departamento de Estratigrafía y Paleontología, Universidad de Granada, 18002 Granada, Spain

[2]Instituto Andaluz de Ciencias de la Tierra, CSIC-Universidad de Granada, 18100 Armilla, Spain

[3]Department of Earth and Environmental Sciences, Ludwig-Maximilians Universität, 80333 Munich, Germany

[4] Institute for Geology, University of Hamburg, 20146 Hamburg, Germany

Supplementary Figures S1 to S7

Species information	Binocular microscope image (0.8x)	Binocular microscope image (2x)
Schizobrachiella sanguinea (Norman, 1868).<br>Depth 3 m, Korčula Island (Croatia, 2008).<br>**Class Gymnolaemata; Order Cheilostomata.**	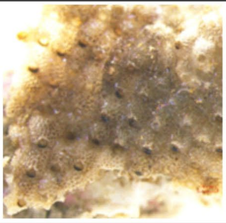	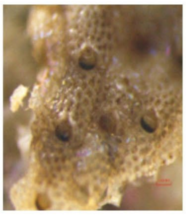
Rhynchozoon neapolitanum (Gautier, 1962).<br>Depth 12 m, Korčula Island (Croatia, 2008).<br>**Class Gymnolaemata; Order Cheilostomata.**	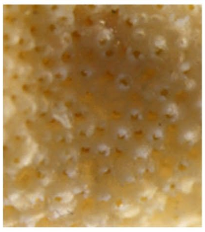	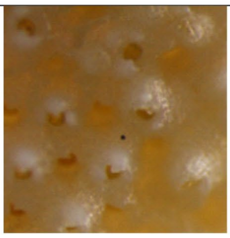
Calpensia nobilis (Esper, 1796).<br>Depth 10 m, Korčula Island (Croatia, 2008).<br>**Class Gymnolaemata; Order Cheilostomata.**	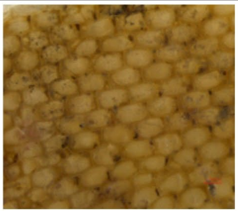	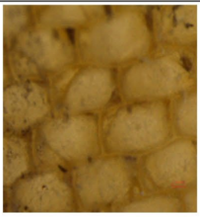
Schizoretepora serratimargo (Hincks, 1886).<br>Depth 12 m, Korčula Island (Croatia, 2008).<br>**Class Gymnolaemata; Order Cheilostomata.**	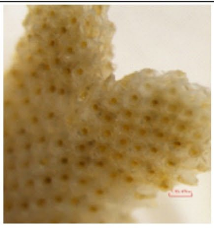	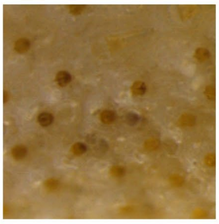
Pentapora fasciallis (Pallas, 1766).<br>Depth 20 m, Korčula Island (Croatia, 2008).<br>**Class Gymnolaemata; Order Cheilostomata.**	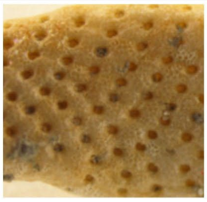	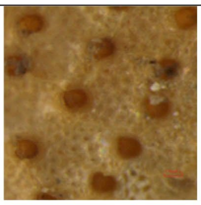
Adeonella pallasii (Heller, 1867).<br>Depth 12 m, Korčula Island (Croatia, 2008).<br>**Class Gymnolaemata; Order Cheilostomata**.	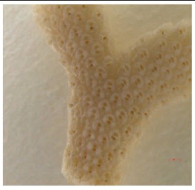	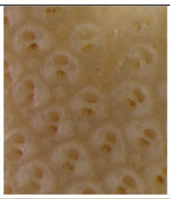
Schizomavella cornuta (Heller, 1867).<br>Depth 20 m, Korčula Island (Croatia, 2008).<br>**Class Gymnolaemata; Order Cheilostomata.**	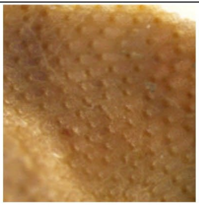	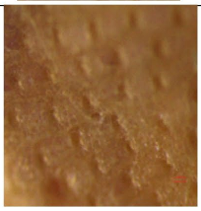
Smittina cervicornis (Pallas, 1766)<br>Depth 20 m, Korčula Island (Croatia, 2008).<br>**Class Gymnolaemata; Order Cheilostomata.**	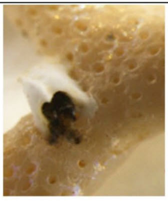	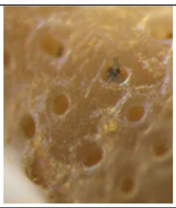

Fig. S1 Summary table of the eight gymnolaemate bryozoans analyzed

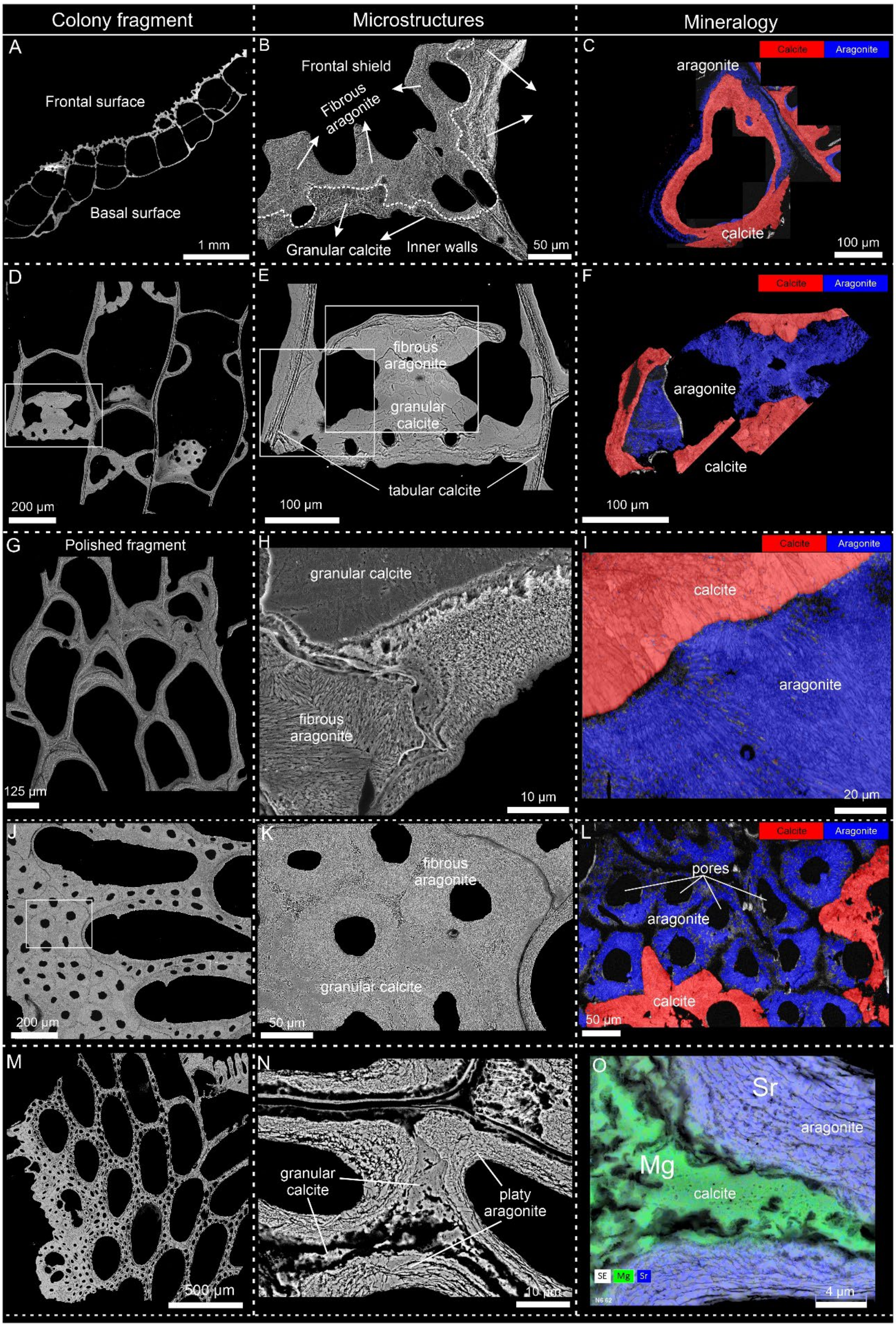

Fig. S2 Microstructures and calcium carbonate polymorph distributions of the bimineralic bryozoans analyzed. A-C. *Schizobrachiella sanguinea*. D-F. *Calpensia nobilis*. H-I. *Schizoretepora serratimargo*. J-L. *Pentapora fascialis*. M-O. *Adeonella pallasii.* The left and intermediate columns are representative SEM images. The right columns are EBSD phase (C, F, I and L) and EDX (O) maps performed in each sample

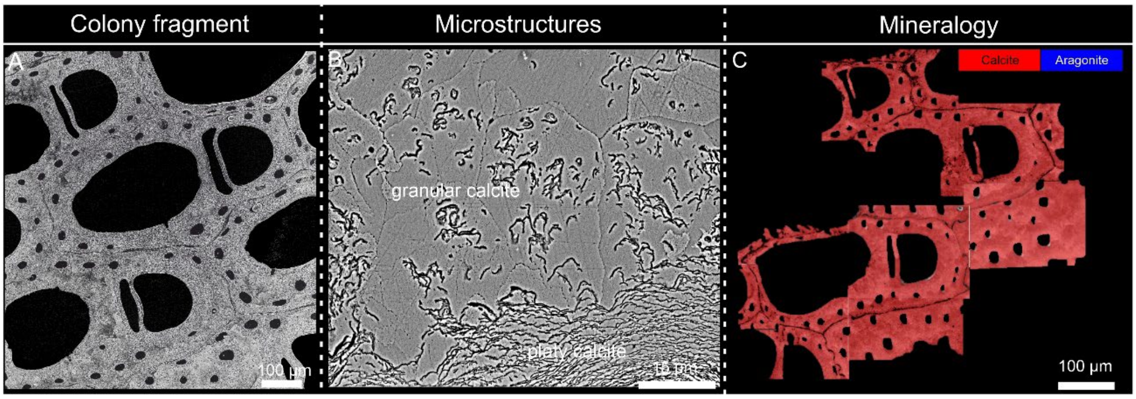

Fig. S3 Microstructures and calcium carbonate polymorph distribution of the fully calcitic bryozoan analyzed, *Smittina cervicornis*. A and B. SEM images of representative areas. C. EBSD phase map

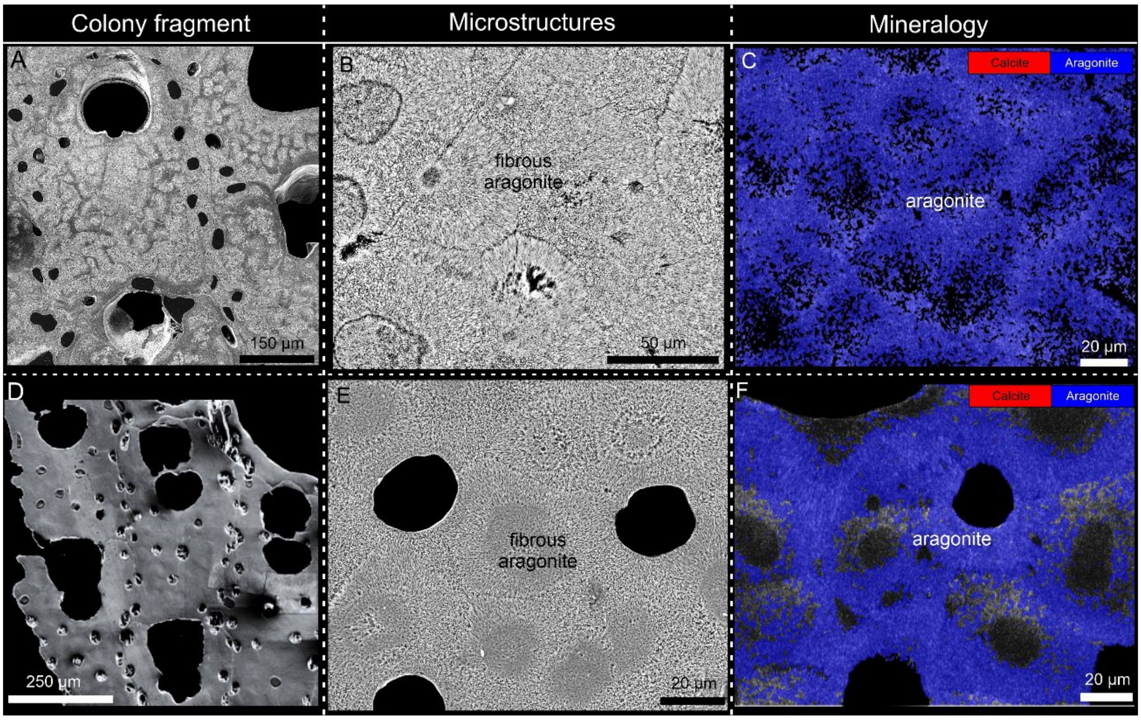

Fig. S4 Microstructures and calcium carbonate polymorph distribution of the aragonitic bryozoans analyzed. A-C. *Rhynchozoon neapolitanum*. D-F. *Schizomavella cornuta.* The left and center columns are SEM images of representative areas. The right columns are EBSD phase maps (C and F)

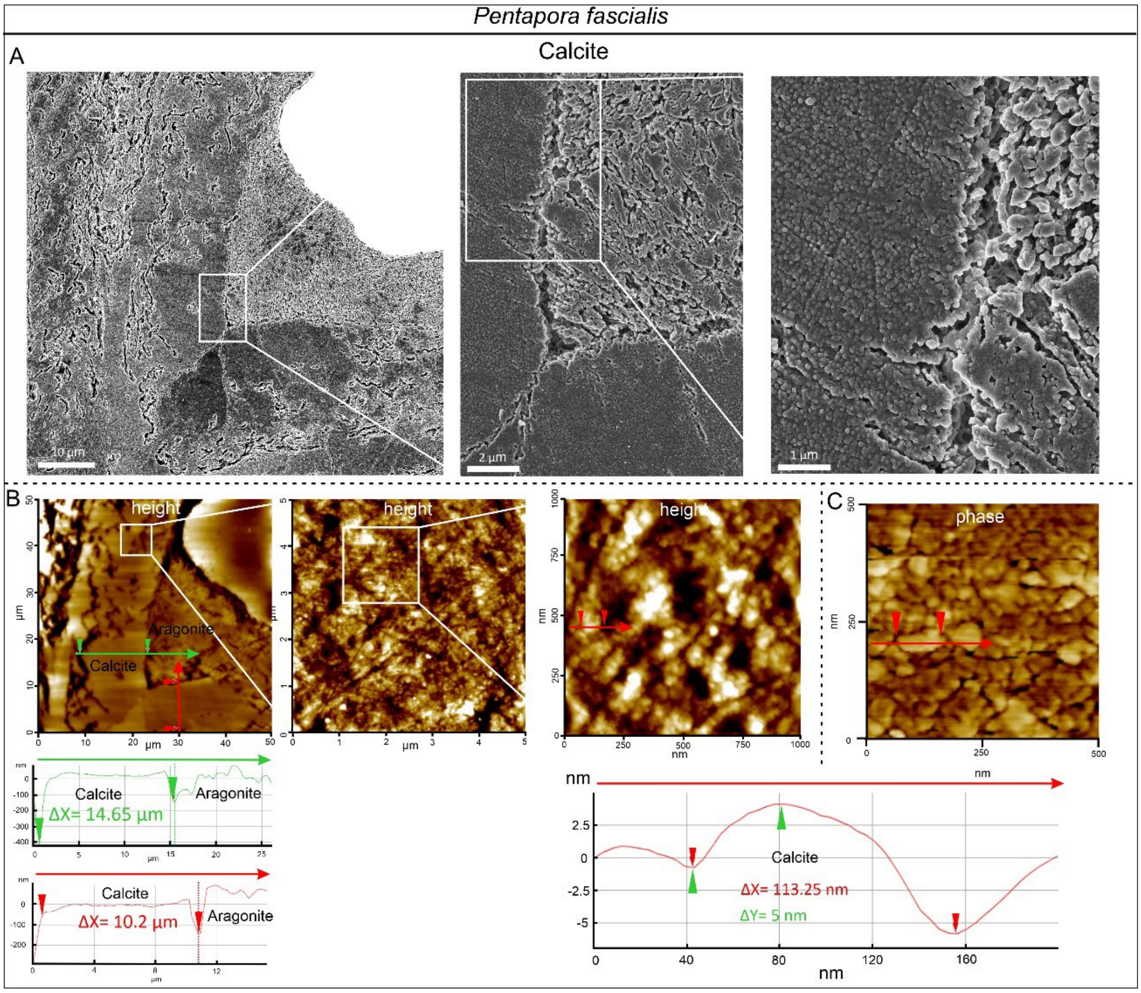

Fig. S5 AFM analysis of the granular calcite of the frontal shield of *Pentapora fascialis*. A. From left to right, SEM images at increasing magnifications of a region similar to that scanned with the AFM. The surface nanoroughness can be appreciated in the right image. B. AFM height images at increased magnifications (framed areas) of the granular calcite. The profiles of the transects outlined in the height images indicate sizes of the calcite grains ranging from 10 µm to 14 µm in width. The selected nano-protrusion has a diameter of 113 nm and a height of 5 nm (see the graphs). C. The phase image shows dark/light contrast between different areas

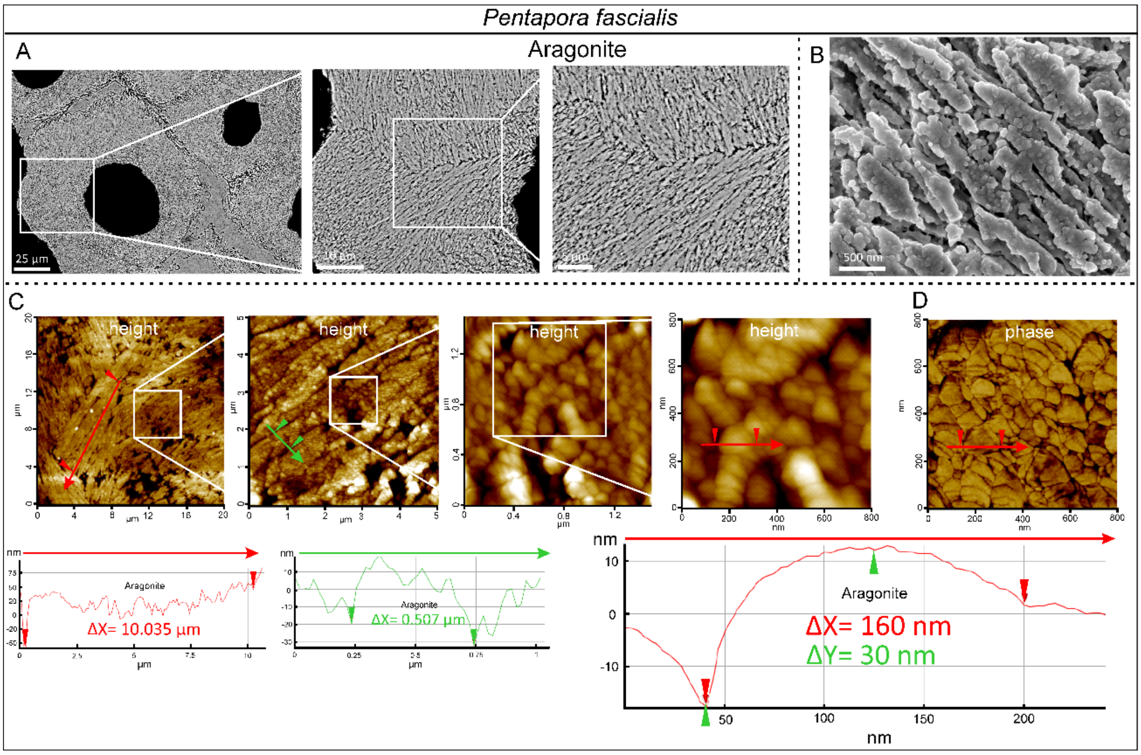

Fig. S6 AFM analysis of the fibrous aragonite that surrounds the pores of the frontal shield of *Pentapora fascialis*. A. From left to right, SEM images at increasing magnifications (framed areas) of a region similar to that scanned with AFM. B. High-magnification view of a set of fibers where the surface nano-roughness can be appreciated. C. From left to right, AFM height images of the fibrous aragonite at increasing magnifications (framed areas). The profiles of the transects drawn at the height images give fiber lengths of 10 µm and close to 0.6 µm in width. The transects of the highest resolution images indicate that the diameter of the selected nano-protrusion is 160 nm and a height of 30 nm. D. The phase map appears homogeneous, indicating no changes in the composition

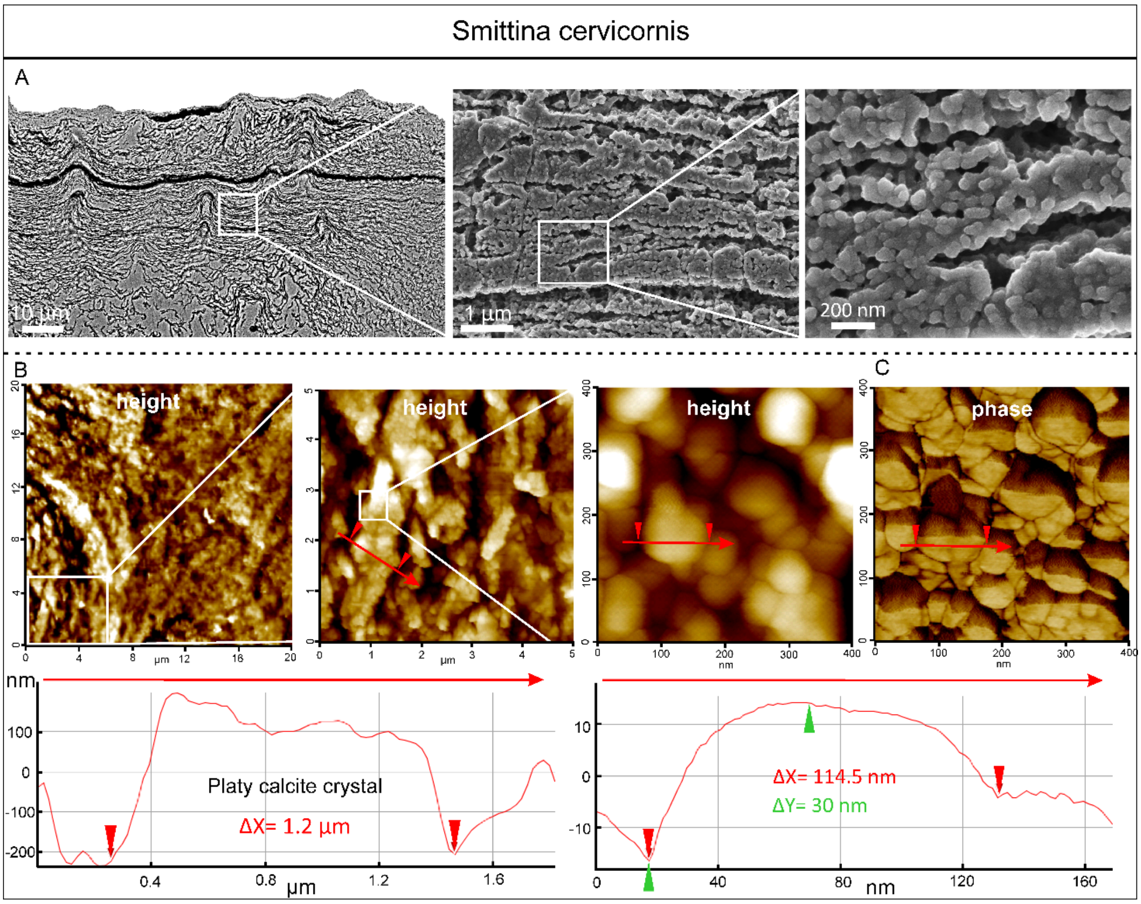

Fig. S7 AFM analysis of the irregularly platy calcite of the external wall of *Smittina cervicornis*. A. From left to right, SEM images at different magnifications (see framed areas) of a similar region to that scanned with the AFM. The surface nanoroughness is clearly visible in the right image. B. AFM height images at increased magnifications (framed areas) of the region scanned. The height profiles indicate a width of the selected calcite plate of 1.2 µm (left) and a diameter of 114.5 nm of the selected nano protuberance, with a height of 30 nm (right). C. Phase image showing the presence of a dark phase at the top edges of the nanoprotrusions
